# Cajal bodies are linked to genome conformation

**DOI:** 10.1038/ncomms10966

**Published:** 2016-03-21

**Authors:** Qiuyan Wang, Iain A. Sawyer, Myong-Hee Sung, David Sturgill, Sergey P. Shevtsov, Gianluca Pegoraro, Ofir Hakim, Songjoon Baek, Gordon L. Hager, Miroslav Dundr

**Affiliations:** 1 Department of Cell Biology, Rosalind Franklin University of Medicine and Science, Chicago Medical School, North Chicago, 60064 Ilinois, USA; 2 Laboratory of Receptor Biology and Gene Expression, Center for Cancer Research, National Cancer Institute, National Institutes of Health, Bethesda, 20892 Maryland, USA; 3 High-Throughput Imaging Facility (HiTIF), Center for Cancer Research, National Cancer Institute, National Institutes of Health, Bethesda, 20892 Maryland, USA

## Abstract

The mechanisms underlying nuclear body (NB) formation and their contribution to genome function are unknown. Here we examined the non-random positioning of Cajal bodies (CBs), major NBs involved in spliceosomal snRNP assembly and their role in genome organization. CBs are predominantly located at the periphery of chromosome territories at a multi-chromosome interface. Genome-wide chromosome conformation capture analysis (4C-seq) using CB-interacting loci revealed that CB-associated regions are enriched with highly expressed histone genes and U small nuclear or nucleolar RNA (sn/snoRNA) loci that form intra- and inter-chromosomal clusters. In particular, we observed a number of CB-dependent gene-positioning events on chromosome 1. RNAi-mediated disassembly of CBs disrupts the CB-targeting gene clusters and suppresses the expression of U sn/snoRNA and histone genes. This loss of spliceosomal snRNP production results in increased splicing noise, even in CB-distal regions. Therefore, we conclude that CBs contribute to genome organization with global effects on gene expression and RNA splicing fidelity.

## Abstract (web summary)

Nuclear bodies can nucleate at sites of active transcription and are beneficial for efficient gene expression. Here, the authors show that Cajal bodies, a prominent type of nuclear body, contribute to genome organization with global effects on gene expression and RNA splicing fidelity.

## Graphical abstract

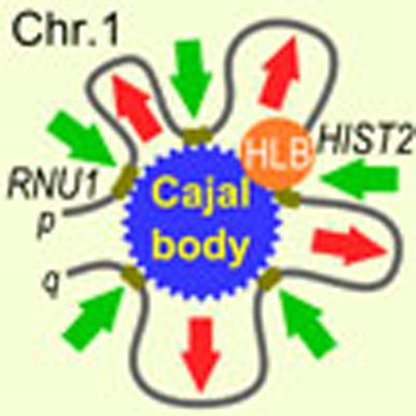

Over the last decade it has become apparent that gene expression is regulated by the dynamic interplay between spatial genome organization and nuclear architecture. Within the membrane-free nuclear interior, chromosomes occupy spatially confined territories that intermingle near their edges^1^,^2^. These territories adopt discrete non-random positions with some chromosomes preferentially situated in the interior and others near the periphery. Moreover, chromatin domains with a similar status of activity are found to cluster into higher order compartments^3^. This can lead to the formation of topological superdomains composed of multiple functionally related genomic loci^4^. In addition, a variety of nuclear bodies (NBs) exist, including the nucleoli, Cajal bodies (CBs), histone locus bodies (HLBs), PML NBs, splicing speckles, paraspeckles and nuclear stress bodies^5^,^6^,^7^,^8^,^9^. Several major NBs have been observed to nucleate at sites of active transcription with highly abundant transcripts or specific genomic regulatory elements functioning as a seeding scaffold in their formation^10^,^11^,^12^,^13^. Hence, these structures may arise by dynamic self-organization after an initial nucleation event, reflecting site-specific activities associated with gene expression and genome maintenance.

It has been suggested that molecular-crowding effects within NBs such as CBs are beneficial for efficient gene expression and NB structural maintenance^14^,^15^. They restrict the reaction volume to promote macromolecular complex assembly and association with substrates^16^. CBs have been implicated in the processing, assembly, modification and final maturation of spliceosomal small nuclear ribonucleic particles (snRNPs) and small nucleolar ribonucleic particles, biogenesis of telomerase, 3′-end processing of histone pre-mRNAs^17^,^18^,^19^, as well as in recycling of spliceosome components after each round of pre-mRNA splicing^20^,^21^.

Previous studies have documented physical associations of CBs with a limited number of major and minor spliceosomal snRNA genes and U3 snoRNA (*SNORD3A*) genes^22^,^23^,^24^,^25^,^26^. The relevance of this finding to snoRNA gene function is uncertain. More than 90% of human U snoRNAs are encoded within the introns of protein-coding and non-coding host genes^27^, whereas the genes encoding the major U3, U8 and U14 snoRNAs (involved in pre-rRNA processing) have autonomous promoters located in intergenic regions. Similarly, some replication-dependent histone genes have been found to be associated with CBs, likely via the physically associated HLB^28^,^29^,^30^. However, complete characterization of the association of CBs with the genome and delineation of the functionally relevant interactions are lacking from existing data.

To understand the nature and extent of CB-proximal genome–genome interactions, we used 4C-seq analysis to characterize genome-wide contacts of CB-associated genes in human cells. Combining 4C analysis with a novel six-colour co-localization DNA FISH imaging and high-throughput automated microscopy screening, we define a genome-wide chromatin interface with CBs. In particular, we identify a complex CB-proximal genome-interaction hub on chromosome 1. Our integrative analysis reveals CB-dependent looping of all categories of sn/snoRNA, small CB-specific RNA (scaRNA) and histone gene loci across the genome into transcriptionally highly active intra- and inter-chromosomal regions. These interactions are greatly reduced in cells where CBs are absent or have been disassembled by specific depletions of essential CB components by RNAi. Small-and total-RNA-seq analyses show critical roles of CBs for the global maintenance of elevated sn/snoRNA expression in CB-containing aneuploidy cells, RNA pol II-driven transcription and splicing that are not limited to CB-proximal genomic regions. Our study demonstrates that CBs contribute to topological chromosome organization and affect the gene expression of target RNAs through proximal association with specific sn/snoRNA gene loci. This ultimately influences RNA maturation processes and splicing fidelity.

## Results

### CB position relative to chromosomes and sn/snoRNA genes

**Figure 1:**
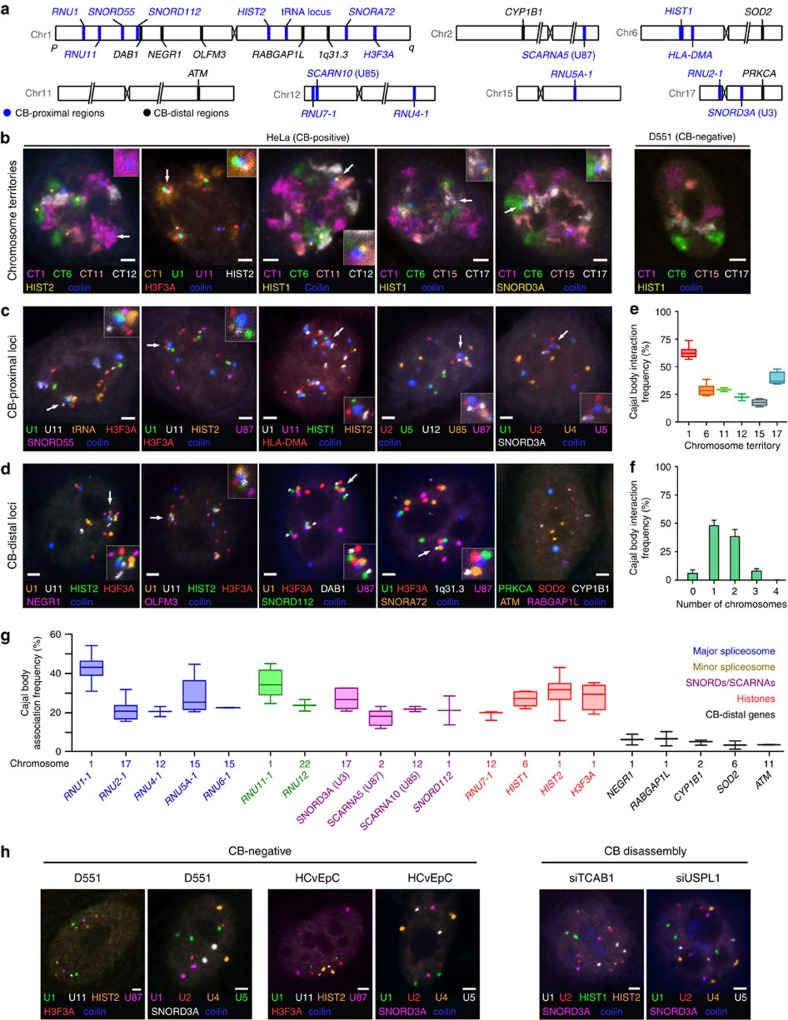
Cajal bodies favourably interact with highly expressed U gene and histone gene loci. (**a**) Approximate chromosomal location of BAC DNA FISH probe target sequences used in this study. Blue text denotes CB-proximal (4C-positive) gene loci and black text denotes non-CB-proximal (4C-negative) gene loci. (**b**) Six-colour fluorescent microscopy analysis of four CTs, CB-interacting gene locus detected by DNA FISH and CB localization visualized by CB marker protein, coilin, in aneuploid HeLa (CB-positive) and primary diploid D551 (CB-negative) cells. Scale bar, 2 μm. (**c**) Six-colour fluorescent microscopy analysis of Cajal body-interacting gene loci by DNA FISH and coilin immunostaining in HeLa cells. (**d**) Six-colour microscopy analysis of CB-interacting and CB-independent negative-control gene loci by DNA FISH and coilin immunostaining in HeLa cells. (**e**) Box and whisker plot of frequencies of CT–CB association interactions (manually scored by proximity to CBs). Error bars depict s.d. and scores represent assessment of at least 150 cells. (**f**) Histogram displaying the frequency of interaction between one CB and (up to four) CTs (manually scored by proximity to CBs). Error bars depict s.d. and scores represent assessment of at least 150 cells. (**g**) Box and whisker plot of frequencies of DNA FISH loci–CB association interactions (manually scored by proximity to CBs). Error bars depict s.d. and scores represent assessment of at least 150 cells. (**h**) Six-colour microscopy analysis of CB-interacting loci by DNA FISH and coilin immunostaining in human primary diploid foreskin D551 cells, human primary diploid cervical epithelial cells (HCvEpC; lacking CBs) and (**i**) HeLa cells following CB disassembly by TCAB1 or USPL1 knockdown.

To increase the number of putative CB-associating gene loci that can be examined by microscopy, we developed a six-colour DNA FISH mapping technique to simultaneously visualize five different gene loci of interest along with coilin, the major CB marker protein, in cells using spectral imaging and linear unmixing (see the ‘Methods' section). We confirmed co-localization of major and minor spliceosomal snRNA genes and histone genes, some of which have previously been documented to be associated with CBs individually^22^,^23^,^24^,^25^ (Fig. 1a–d). We simultaneously visualized CBs, several gene loci and four chromosome territories (CTs) which harbour major and minor spliceosomal snRNA and prominent sn/snoRNA genes. The majority of CBs were frequently located at the periphery of CTs, where two or more chromosomes interact with each other (Fig. 1b,e,f). The most frequent CTs associated with CBs are CT1 (62% of CTs associated with CB) followed by CT17 (38%), CT6 (31%) and CT11 (31%; Fig. 1g). After spatial analysis of the two-dimensional (2D)-projected nuclear and CT regions, we calculated the theoretical random CT1–CB association frequency to be 23.5%. This is considerably lower than the observed frequency (62%), indicating the specificity of this interaction and the significant role of chromosome 1 in CB formation. CBs are predominantly associated with one CT (48%) or two CTs (40%), and less often with three CTs (10%; Fig. 1f). In agreement, CBs are associated with one (46%), two (28%) or three genomic regions (9%) located on different chromosomes. These data indicate that CBs form frequent inter-chromosomal gene clusters with two or three chromosomes.

As an exception to this pattern, one CB, and occasionally two CBs, was frequently found to be predominantly localized adjacent to chromosome 1 in HeLa cells. The uniquely identifiable CB was termed ‘intra-chr1 CB'. To further investigate the role of chromosome 1 in CB formation and function, we visualized many loci throughout chromosome 1 by six-colour DNA FISH. Several highly expressed small U RNA genes and the histone gene clusters (*HIST2* and *H3F3A*) from different arms of the chromosome were frequently found to be associated with the intra-chr1 CB (Fig. 1c). Quantification of DNA FISH revealed that *RNU1* (1p36), encoding the U1 snRNA gene array^31^, is the locus most frequently associated with the CB (Fig. 1g, median association allelic frequency 42.7±1.1% (*N*=2), similar to a previous report on HeLa cells^10^). Other highly expressed U snRNA and snoRNA genes residing on both arms of chromosome 1, including RNA components of the major and minor spliceosomal complexes, two histone gene clusters (*HIST2* and *HF3FA*) and other intron-encoded genes (*SNORD*, *SNORA* and *SCARNA*), also display significant CB association frequencies (ranging from 35 to 23%). Importantly, other genes in sn/snoRNA-poor regions, but which display expression of *NEGR1* and *RABGAP1L* on chromosome 1, were found to have association frequencies significantly lower (in the range of 6–8%), suggesting that these gene–CB interactions are selective (Fig. 1d,g). We tested specific association of CBs with expressed sn/snoRNA genes by visualizing two intron-encoded snoRNA loci *SNORD112*, located on the p-arm (35 Mbp), and *SNORA72*, located on the q-arm (185 Mbp) of chromosome 1, as well as with neighbouring U RNA gene-poor regions. The CB association frequencies of *SNORD112* and *SNORA72* were 21.2 and 32.4%, respectively, while their neighbouring loci, *DAB1* (40 Mbp) and 1q31.3 (177 Mbp), had association frequencies of 9.8 and 6.1%, respectively (Fig. 1a; all bp coordinates in hg19; Fig. 1g and Supplementary Fig. 1b).

Quantification of CB association of sn/snoRNA genes on other chromosomes, including *SCARNA5/10* and *SNORA25* (Fig. 1g and Supplementary Fig. 1b), suggested that CB-U RNA gene clustering may be a genome-wide trend. The association frequency of CBs with sn/snoRNA loci on several chromosomes ranged from 12 to 20%, with the exception of one genomic region on the p-arm of chromosome 17 which had a CB association frequency similar to *RNU1* (42.3±3.8%, *n*=2). This particularly high association may reflect the high-frequency simultaneous association of CBs with chromosome 1 and chromosome 17 (Fig. 1e). Negative control genes on other chromosomes (*CYP1B1* on chr2, *ATM* on chr11, *PRKCA* on chr17) displayed an even lower association frequency with CBs than those for chromosome 1 (median 4–5%; Fig. 1g and Supplementary Fig. 1b).

Since CBs have been reported to physically associate with the HLBs, which regulate expression and the unique 3′-end pre-mRNA processing of histone genes in a cell-cycle-dependent manner, we sought to distinguish specific histone gene clusters involved in co-clustering^12^,^29^. We co-visualized CBs together with the major and minor histone clusters, *HIST1* and *HIST2*, and HLBs by the histone gene transcription factor NPAT^32^. The major histone cluster, *HIST1* on chromosome 6, co-localized primarily with a HLB, whereas the minor histone cluster, *HIST2* on chromosome 1, was mostly associated with a CB and in some instances with a smaller HLB (Supplementary Fig. 1d). In addition, we observed an association between another histone locus *H3F3A*, encoding the replication-independent histone variant H3.3, located on the distal end of the q-arm of chromosome 1, and *RNU1* in proximity of the intra-chr1 CB (Fig. 1c,g). These data indicate that HLBs preferentially form around the major *HIST1* locus and other histone loci are responsible for targeting the intra-chr1 CB where smaller HLBs form (Supplementary Fig. 1d).

### CB depletion coincides with altered gene positioning

To test whether CBs actively contribute to clustering of genes, we induced the disassembly of CBs in HeLa cells by knocking down either TCAB1 or USPL1, two distinct but essential CB components. TCAB1 (also known as WRAP53 or WDR79) has been identified as a component of CB-specific scaRNAs and telomerase and is responsible for recruitment of the SMN complex to CBs^33^,^34^. SUMO isopeptidase USPL1 has also been reported as a key CB-localized factor involved in transcription of snRNA genes^35^,^36^. When CBs were perturbed by siRNA knockdown of TCAB1 or USPL1, we could no longer observe the spatial clustering of previously examined sn/snoRNA loci and the histone loci (Fig. 1h). A similar pattern was observed when we analysed primary diploid cells that lack CBs (D551, Fig. 1b,h). Furthermore, we used a previously published scoring system^37^ to quantify the relative change in position of major histone gene cluster HIST1 within the chromosome 6 territory after CB disassembly (Supplementary Fig. 1e–h). In CB-depleted HeLa cells, the distribution of *HIST1* was altered and was preferentially located within chromosome 6 (21.28 versus 48.02%) compared with the periphery of the territory (29.36 versus 16.95%) consistent with downregulation of histone genes after CB disassembly. These data strongly indicate that numerous sn/snoRNA and histone loci located across the genome associate with CBs and may require the presence of CBs to maintain their spatial organization and high expression status.

### CB position mediates Chr1 topology and sn/snoRNA levels

**Figure 2:**
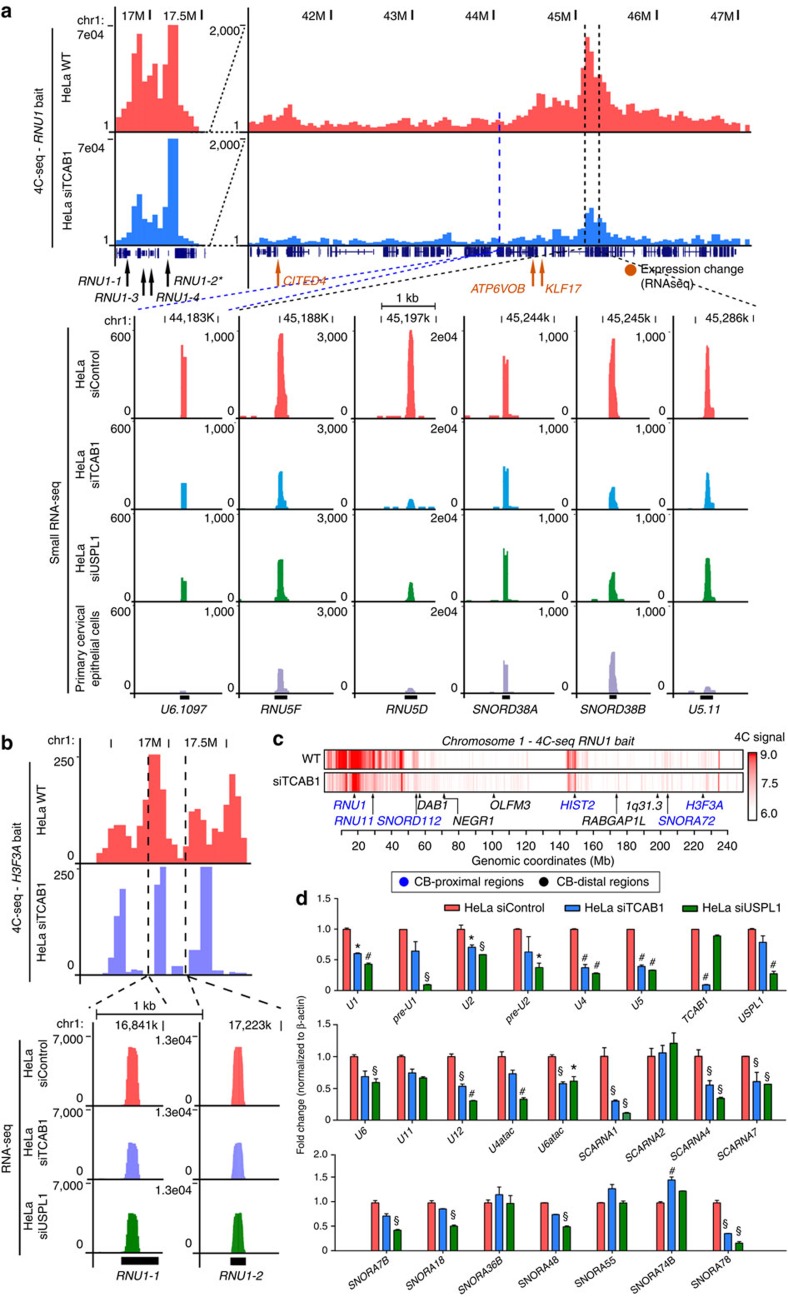
Chromosome 1 undergoes a substantial topological rearrangement following Cajal body disassembly. (**a**) Representative 4C-seq contact profile of intra-chromosomal, CB-dependent interactions using a *RNU1* bait sequence for siControl (HeLa WT, pink) and siTCAB1 (TCAB1kd, blue) treatments. A number of expressed U genes were enriched within this region (dotted lines), which were determined to decrease following siTCAB1 and siUSPL1 (green) treatment by small RNA-seq. The expression of these genes within human primary cervical epithelial cells is also shown (purple). The bait region is depicted between 16.5 and 17.5 MBp (RNU1-2*). Several genes of interest were also located in this region, denoted by arrows (orange=RNA-seq expression change following TCAB1 or USPL1 knockdown). The *y*-axis displays the sequencing read counts/genome fragment, (**b**) representative 4C-seq contact profile of *H3F3A* bait to the *RNU1* array region on chromosome 1 in control (pink) and siTCAB1-treated (blue) in HeLa cells. RNA-seq expression profile of *RNU1-1* and *RNU1-2* loci within this genomic region (dotted lines) show a sensitivity of these U genes to CB disruption by TCAB1kd or USPL1kd (green) by small RNA-seq. *y*-axis displays the read counts/genome fragment, (**c**) chromosome 1 *RNU1* 4C-seq physical contact profile (red, log10 scale) in HeLa cells under normal conditions (WT) following CB disassembly using TCAB1 siRNA. Locations of CB-proximal (blue) and -distal regions (black) used for selection of BAC probes are also indicated. (**d**) Validation of U RNA gene and small nucleolar RNA (SNORD/SNORA/SCARNA) expression changes in HeLa cells following siControl, siTCAB1 or siUSPL1 treatment normalized to β-actin (as depicted). *n*=2, *P<0.05, ^#^*P*<0.01, ^§^*P*<0.001 compared with HeLa siControl, significance was assessed by Student's *t*-test. Error bars represent s.e.m.

**Figure 3:**
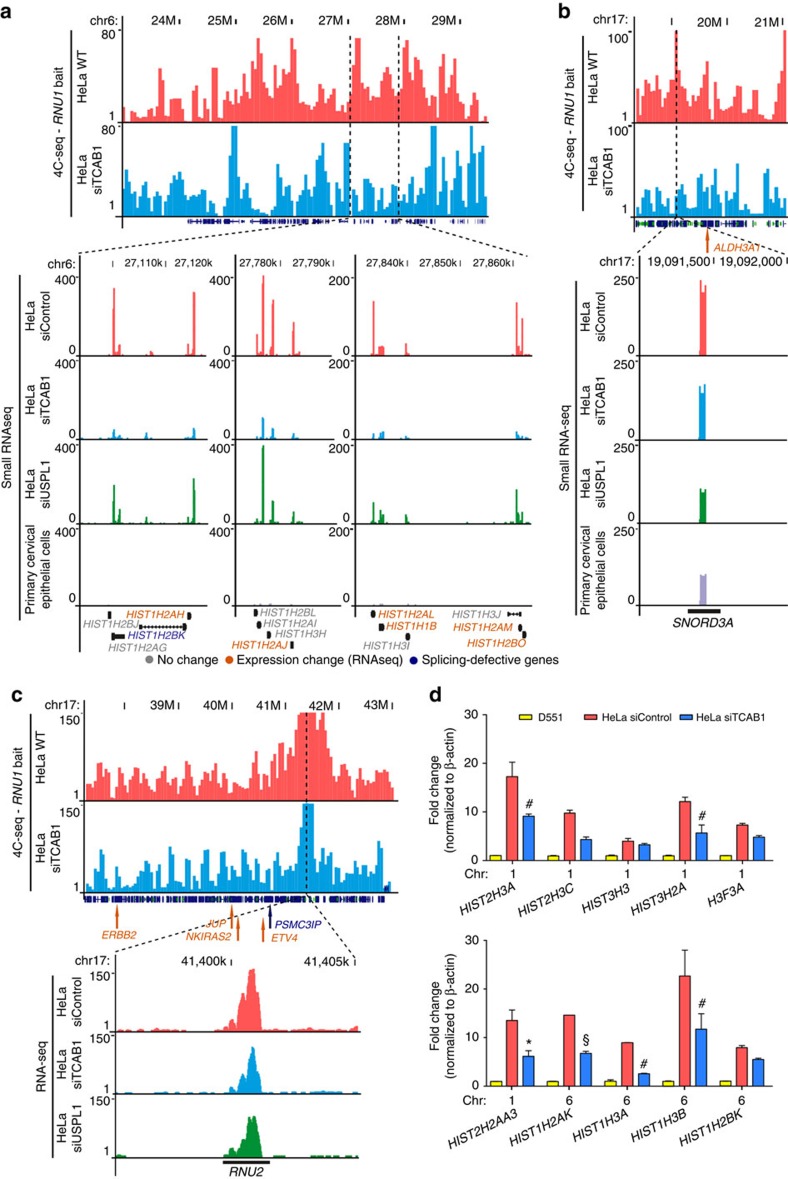
CB-mediated inter-chromosomal interactions influence histone and U RNA gene expression. (**a**) Representative 4C-seq contact profile of inter-chromosomal, CB-dependent interactions using a *RNU1* bait sequence (chromosome 1) for HeLa WT (pink) and siTCAB1 (blue) treatments at the *HIST1* locus on chromosome 6. A number of expressed histone genes are enriched within this region (dotted lines) which were determined to decrease following siTCAB1 and siUSPL1 (green) treatment and poorly expressed in primary cervical epithelial cells (purple) by small RNA-seq. *y*-axis displays the read counts/genome fragment. (**b**,**c**) 4C-seq contact profile for the *SNORD3A* (U3 snoRNA, **b**) and *RNU2* (U2 snRNA, **c**) loci on chromosome 17 using a RNU1 bait in HeLa cells. Control (HeLa WT, pink) and siTCAB1 (blue) are shown, as well as small RNA-seq profile for *SNORD3A* in control, siTCAB1, siUSPL1 (USPL1kd, green) and primary cervical epithelial cells (purple). *y*-axis displays the read counts/genome fragment. Several genes of interest were also located in this region, denoted by arrows (orange=RNA-seq expression change following TCAB1 or USPL1kd, blue=splicing defect detected, grey=no change). (**d**) Validation of a selection of histone genes by qRT–PCR normalized to β-actin (yellow=primary D551 cells, red=HeLa siControl, blue=HeLa siTCAB1). *n*=2, *P<0.05, ^#^*P*<0.01, ^§^*P*<0.001 compared with HeLa siControl, significance was assessed by Student's *t*-test. Error bars represent s.e.m.

Given the evidence for CB-proximal positioning of U sn/snoRNA and histone genes from DNA FISH, we sought to map the full spectrum of CB-proximal genomic regions using the chromosome conformation capture technique^38^ followed by high-throughput sequencing (4C-seq) in HeLa cells (Figs 2 and 3). As the bait locus we selected *RNU1* which exists as a highly expressed multi-copy gene array on chromosome 1 (1p36), and has the highest CB contact frequency among genes analysed in our six-colour DNA FISH analysis. As a complementary bait we chose the replication-independent histone gene *H3F3A* (1q42.12) encoding the histone variant H3.3 (ref. 39) on chromosome 1, which is constitutively transcribed and frequently associated with CBs in close proximity to *RNU1* (Fig. 1c). *Hin*dIII 4C libraries were constructed in replicates and sequenced to obtain the contact profiles in HeLa cells (Supplementary Fig. 3a,b). Replicate reproducibility was confirmed, and pooled 4C data were used for subsequent analyses (Supplementary Fig. 3c). The majority of the 4C signal indicated intra-chromosomal interactions but some distal inter-chromosomal interactions were also detected. The *RNU1* 4C profile in HeLa revealed that the contact loci interacting with the intra-Chr1 CB through *RNU1* are enriched with all classes of U RNA genes (*RNU*, *SNORD*, *SNORA*, *SCARNA*, *snoU* and U1 snRNA class I pseudogenes on 1q12–21; *P* value<0.01; see the ‘Methods' section and Fig. 2a). For example, a strong interaction with *RNU1* was observed for the U11 snRNA (*RNU11*) gene (Supplementary Fig. 2a) located 12 Mb downstream, as well as for *HIST2* and *H3F3A* on the other arm of chromosome 1 (Fig. 2c), consistent with the DNA FISH data.

To identify CB-dependent genome organization, we performed 4C analysis after disassembly of CBs using siRNA targeting TCAB1 (siTCAB1; Fig. 2, Supplementary Data 1). To understand the CB-dependent organization in terms of sn/snoRNA expression, we also performed small RNA-seq on HeLa cells treated with siTCAB1, siUSPL1 or siControl. The intra-chromosomal interactions observed in control cells were globally reduced in CB-disrupted cells, including the chromosome end-to-end interaction between *RNU1* and *H3F3A* (Fig. 2b,c). In parallel, a substantial fraction (11%) of small RNA genes had a reduced expression in either knockdown (177 and 175 loci for siTCAB1 and siUSPL1, respectively), while relatively few small U RNAs showed an increased expression after knockdown (3–4%; Supplementary Data 2). Both major and minor spliceosomal U snRNA genes were downregulated following CB disassembly. The expression of these U RNA genes was typically much higher in HeLa cells (cervical carcinoma) compared with a tissue-matched control, human primary diploid cervical epithelial cells (Fig. 2a). In fact, small RNA-seq profiling revealed an astonishing range of expression for the diverse sn/snoRNA genes, spanning eight orders of magnitude. Most of the small RNA genes interacting with *RNU1* belong in the highest expression category (Supplementary Data 2). We noted differential behaviours of sn/snoRNAs in CB-disrupted cells based on their status in the control primary cells. Following CB disassembly, the *SNORA* and *SNORD* genes that are highly expressed in the control and HeLa cells showed no major changes in expression. However, U sn/snoRNAs with low expression in the control cells but high in HeLa, such as the *RNU1* array (*RNU1-1*, *RNU1-2*) and *RNU11*, had markedly decreased expression even in the same contact domain (Fig. 2c and Supplementary Fig. 2a). We confirmed CB-dependent expression of sn/snoRNAs observed in small RNA-seq, including all the major and minor spliceosomal snRNA components, by qRT–PCR (Fig. 2d). Nascent extended pre-processed U1 and U2 RNA (pre-U1 and -U2) expression was decreased after CB disassembly indicating that CBs function as important transcriptional initiation sites for U RNA genes. No decreases in U RNA gene expression were observed after TCAB1kd in D551 primary diploid cells that lack CBs (Supplementary Fig. 2b). Therefore, we conclude that the observed reduction in U RNA gene expression levels is a result of CB disassembly and not secondary effects of our TCAB1 or USPL1 depletion.

### Many inter-chromosomal interactions of *HIST1* are CB proximal

In addition to the CB-dependent intra-chromosomal interactions of *RNU1*, 4C analysis indicated decreases in intra-chromosomal changes following disassembly of CBs (Figs 2 and 3, and Supplementary Fig. 3). Consistent with the idea that CBs may influence functional genome organization, enrichment for sn/snoRNA genes within contact regions from both *RNU1* and *H3F3A* 4C was reduced in the absence of CBs (permutation test; see the ‘Methods' section). Although detection of inter-chromosomal contact dynamics is technically challenging with 4C, interaction of *RNU2* (ref. 40) and *SNORD3A* (U3), both on chromosome 17, with *RNU1* depended on CBs (Fig. 3b,c), and their expression was notably diminished in CB-disrupted cells.

Histone gene clusters on chromosomes 1 and 6 also had CB-dependent interactions with both *RNU1* and *H3F3A* loci. In particular, *HIST1* on chromosome 6 contains over fifty replication-dependent histone genes. Similar to sn/snoRNAs, the expression of *HIST1* genes, highly abundant in HeLa cells, was suppressed after CB disassembly (Fig. 3a,d). This can likely be ascribed to the concomitant loss of the association of HLBs with disassembled CBs. These results suggest a role of CBs in genome organization that reaches beyond chromosome 1, promoting the physical clustering of sn/snoRNAs and histone loci and their robust expression.

### High-content DNA FISH CB co-localization analysis

**Figure 4:**
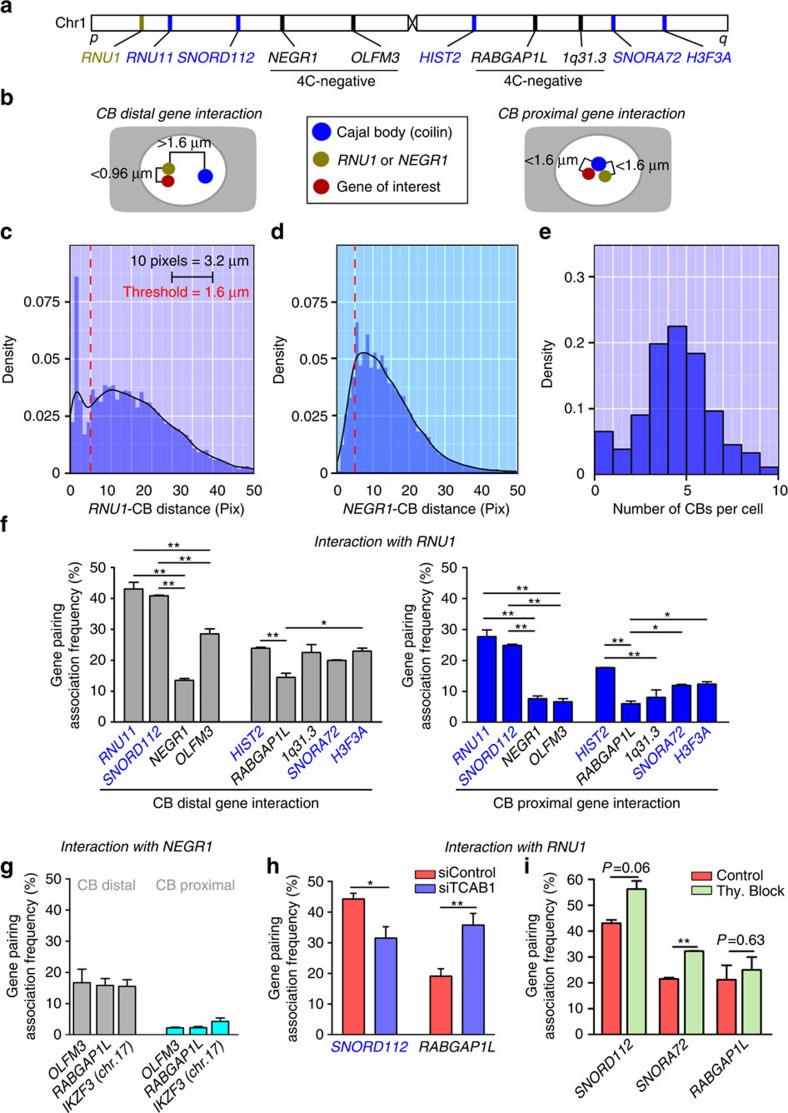
High-content microscopic validation of Cajal body-dependent gene interactions. (**a**) Approximate chromosomal location of BAC DNA FISH probe target sequences used in automated high-throughput DNA FISH-IF co-localization studies. Blue text denotes CB-interacting (4C-positive) loci. Regions determined to be non-CB interacting are denoted by (4C-negative, black text). (**b**) A schematic detailing the threshold criteria used to determine whether a gene–gene cis-interaction or gene–CB interaction was meaningful. The *RNU1* gene and various gene locus of interest located on chromosome 1 were determined to interact in the absence of CBs when distance was >5 pixels from a CB, but ≤3 pixels between spot centres (CB-independent). CB-clustered gene loci were determined to occur within a distance of ≤5 pixels between the CB spot centre and the *RNU1* loci spot centre (CB-dependent). Red spot=*RNU1*, green=variable gene of interest, purple=CB. (**c**) Histogram of the per-*RNU1* FISH spot minimum distances between *RNU1* and CB (in pixels, 10 pixels=3.2 μm). Blue bars=binned data, black line=estimated density function, red dotted line=threshold criteria (1.6 μm). (**d**) Histogram of the per-CB spot minimum distances between *NEGR1* and CB (in pixels, 10 pixels=3.2 μm). Blue bars=binned data, black line=estimated density function, red dotted line=threshold criteria (1.6 μm). (**e**) Histogram depicting the number of CBs per cell in HeLa cells as quantified by high-throughput imaging (>1,000 cells). (**f**) Association frequency between *RNU1* and various genes of interest on chromosome 1 (plotted in order relative to *RNU*1) as determined by automated high-throughput imaging. Interactions were grouped according to the criteria detailed in **b**. (**g**) Comparisons were also made between sn/snoRNA-poor loci. Total gene pairing was also assessed after (**h**) siTCAB1 treatment and (**i**) cell cycle arrest by double-thymidine treatment (Thy. Block). *n*=2 (>1,000 cells per replicate), error bars depict s.e.m., *P<0.05, **P<0.01, ***P<0.001 versus *RNU1-NEGR1* or *RNU1-RABGAP1L* was assessed by Student's *t*-test. Error bars represent s.e.m. CB-distal interactions=grey, CB-dependent interactions=dark blue. Red=siControl, blue=siTCAB1, light green=double-thymidine block.

Interactions captured by 4C rely on high contact frequency between *RNU1* and the intra-chr1 CB and do not directly establish the interactions between CB and the *RNU1* contact loci^41^. To quantitatively assess how often *RNU1* cis-contacts are co-localized with the intra-chr1 CB, we used automated high-throughput microscopy of DNA FISH/IF co-localization patterns (see the ‘Methods' section and Fig. 4). Following data acquisition from >1,000 cells per locus pair and automated nuclear segmentation and spot detection of DNA FISH and IF signals, we analysed the minimum pairwise Euclidean distances and classified co-localizations based on whether they occur near a CB (Fig. 4b). *RNU1* displayed a preferential association with CBs, confirming our earlier DNA FISH data (Fig. 4c). When several 4C loci were probed along chromosome 1, *RNU1* contact frequency for all of the 4C-positive loci was significantly higher compared with the 4C-negative control locus located on the same chromosome arm (Fig. 4d). Our high-throughput approach detected some of the gene co-localizations involving *RNU1* occurring away from CBs (Fig. 4f, ‘CB-distal gene interaction'), which likely correspond to stochastic predecessors of CB-associated gene clusters. These interactions occurred as frequently as gene-pairing events between *NEGR1* and other sn/snoRNA-poor regions. Importantly, the *RNU1* association frequency profile over all the examined loci was independent of whether they were proximal to CBs or not (Fig. 4f and Supplementary Fig. 4a,b) and greater than the CB-proximal pairing frequency displayed by *NEGR1* and other sn/snoRNA-poor loci (Fig. 4g, Supplementary Fig. 4c). Disassembly of CBs by TCAB1kd resulted reduced the association frequency between *RNU1* and *SNORD112* (two CB-proximal gene loci) but increased the frequency of gene pairing between *RNU1* and *RABGAP1L* (sn/snoRNA-deficient) confirming our hypothesis that CBs are an important genome organizer in HeLa cells (Fig. 4h). These phenomena were not due to previously reported changes in cell cycle progression,^42^ as gene pairing between *RNU1* and other sn/snoRNA-dependent regions, but not a sn/snoRNA-poor region, was increased on induction of a cell cycle block by double thymidine at the G1/S border (Fig. 4i). In addition, expression of a number of highly sensitive U RNA and sn/snoRNA genes was largely unaffected by cell cycle perturbation (Supplementary Fig. 4f)

### CB depletion induces limited global transcriptional effects

**Figure 5:**
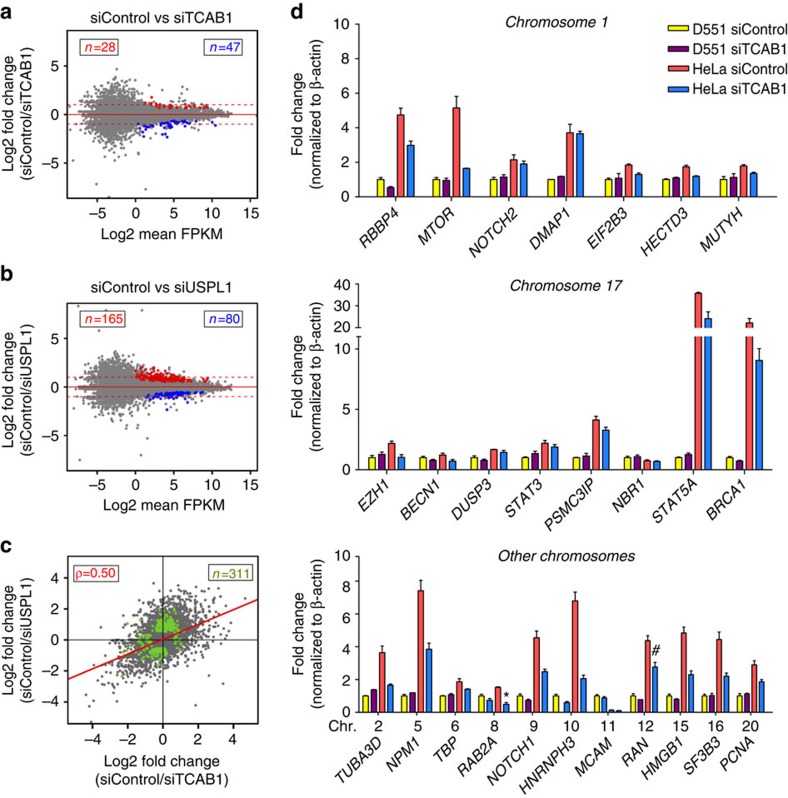
Differential gene-level transcription and splicing following Cajal body disruption. (**a**,**b**) Ratio versus mean abundance scatterplots for the siTCAB1 (**a**) and USPL1 (**b**) treatments. Coloured dots represent gene differentially expressed (at FDR-adjusted *P* value<0.01, red=higher in siControl, blue=higher in knockdown). (**c**) Ratio/Ratio scatterplot of fold-change differences in either siUSPL1 or siTCAB1 treatments. Dots in green have adjusted *P* value<0.01 in either comparison. Dots in the lower left and upper right quadrants indicate fold changes in the same direction. (**d**) Validation of a selection of genes within 4C contact regions by qRT–PCR normalized to β-actin (yellow=primary D551 cells, red=HeLa siControl, blue=HeLa siTCAB1). *n*=2, *P<0.05, ^#^*P*<0.01, ^§^*P*<0.001 compared with HeLa siControl, significance was assessed by Student's *t*-test. Error bars represent s.e.m.

To examine the role of CBs in transcription, we analysed the expression profiles from RNA-seq performed on CB-depleted HeLa cells using siRNA-mediated knockdown of TCAB1 or USPL1. We found that gene expression profiles in both knockdowns and siControl HeLa were similar (*r*=0.99), indicating that the global effects of CB disassembly on transcription are modest (Fig. 5a–c). We confirmed the RNA-seq results by qRT–PCR, validating that CB disassembly induces various but limited expression changes of other Pol II-dependent genes within 4C contact regions (Fig. 5d). Despite the requirement for CBs in sn/snoRNA gene transcription, we found no significant transcriptional changes of host genes whose introns encode U snoRNAs in 4C contact regions. However, we identified 259 genes, showing differential expression in knockdown versus siControl (Supplementary Data 3), and intersected these genes with *RNU1* and *H3F3A* 4C data. We found that only 87 genes (34%) residing within 50 kb of 4C-positive contact regions, and the majority (66%) of the mis-regulated genes were farther away, suggesting that the transcriptional effect reaches beyond the genes in immediate physical proximity of CBs. Among the genes within 50 kb of 4C contact regions, several involved in splicing (for example, *SNRPA1, PRPF3, SMC1A*, *GEMIN5*) were differentially expressed. In addition, multiple histone genes from histone cluster 1 (*HIST1*) and 2 (*HIST2*) displayed expression changes. A summary of CB-dependent genes and their ontology is shown in Supplementary Fig. 6.

Together with the small RNA-seq data indicating that the disassembly of CBs induces a significant curtailment of U RNA gene expression, the CB also contributes to high expression levels of key spliceosomal components, such as *SNRPE* (Sm protein E) and *SNRPA* (U1 snRNP-specific protein A) but may also be required for their sustained expression. This is in accordance with the role of CBs as a catalytic processing site for spliceosome reassembly.

We also observed potential feedback control mechanisms between CB components. CB disruption by specific knock-down conditions invoked compensatory upregulation of *COIL*, the gene encoding coilin, the critical self-interacting structural CB component, and Gemin5, another CB component of the SMN protein complex. In contrast, the gene encoding the Integrator subunit S3 (a member of the Integrator complex responsible for the 3′-end processing of nascent extended pre-U snRNA transcript^43^,^44^), *INTS3*, which directly interacts with coilin and is able to nucleate CBs *de novo*, is downregulated.

### CB disassembly coincides with altered RNA processing

**Figure 6:**
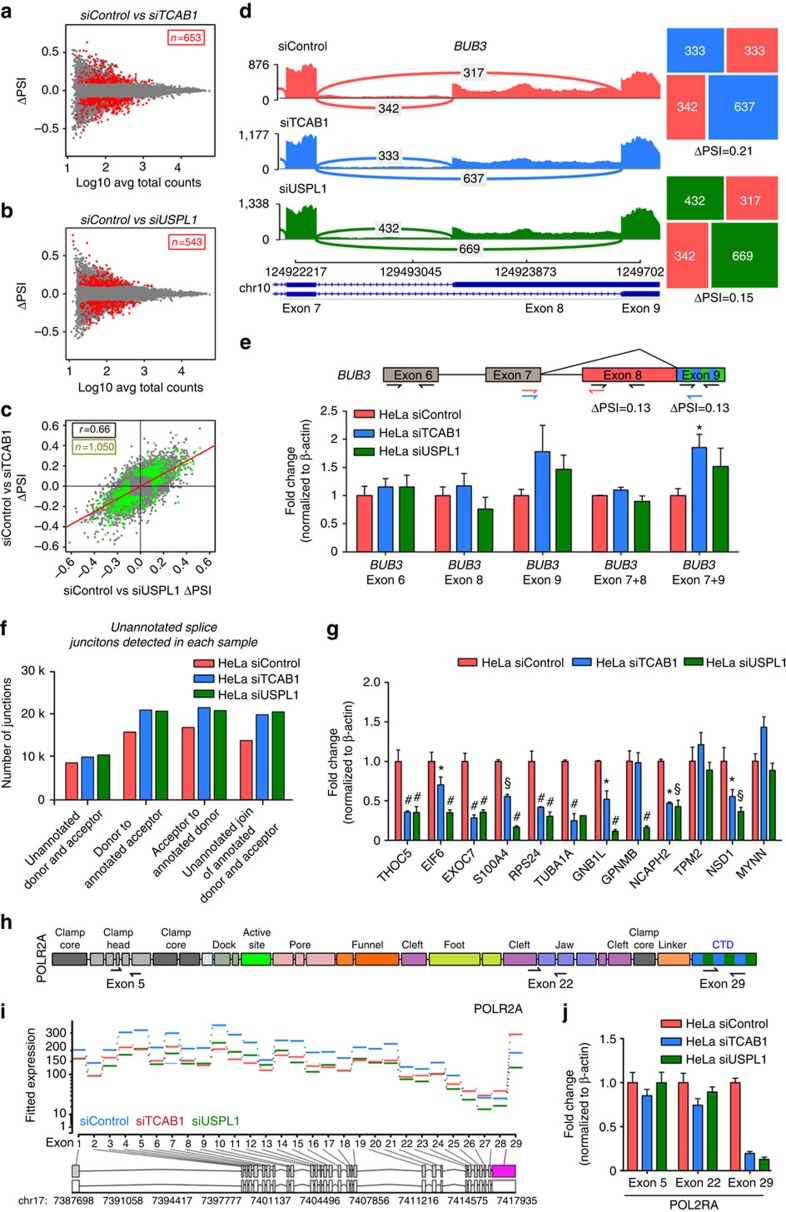
CB depletion increases unannotated RNA splicing including RNA polymerase II CTD deletion. (**a**,**b**) Difference in percentage spliced in (Delta PSI) versus mean abundance (log10 average total read counts) scatterplots for siTCAB1 (**a**) and siUSPL1 (**b**) treatments. Coloured dots represent local pairwise splicing events that are differentially spliced (at FDR-adjusted *P* value<0.01, red=higher in siControl, blue=higher in knockdown). (**c**) Delta PSI/Delta PSI scatterplot of splicing differences in siUSPL1 or siTCAB1 treatments. Dots in green have adjusted *P* value=0.01 in either comparison. Dots in the lower left and upper right quadrants indicate fold changes in the same direction. (**d**) Sashimi plot of RNA-seq coverage over exons and junctions in the BUB3 locus. Differential splicing of an alternative acceptor is indicated. Squares are mosaic plots of splice junction counts within this splicing event. (**e**) Validation of alternative splicing of BUB3 detected by DEXseq through qRT–PCR in HeLa cells treated with siControl (red), siTCAB1 (blue) or siUSPL1 (green). Primers detected exon 6, 8, 9, 7+8 or/and +9 (as depicted). *n*=2, significance was assessed by Student's *t*-test. Error bars represent s.e.m. (**f**) Distribution of splice junctions detected in HeLa cells exposed to siControl, siTCAB1 or siUSPL1 (blue, red and green, respectively). (**g**) Validation of alternative splicing of a selection of genes detected by DEXseq through qRT–PCR in HeLa cells treated with siControl (red), siTCAB1 (blue) or siUSPL1 (green). *n*=2, *P<0.05, ^#^*P*<0.01, ^§^*P*<0.001 compared with HeLa siControl, significance was assessed by Student's *t*-test. Error bars represent s.e.m. (**h**) Location of primer sequences recognizing exons 5, 22 (both non-CTD) and 29 (CTD tail) within the gene encoding the largest subunit of RNA polymerase II (POL2RA), which were used to validate a previously unannotated alternative splicing mechanism for POL2RA. (**i**) DEXseq results for the POL2RA locus, indicating significant differential expression of exon 29, encoding the CTD tail under TCAB1 (blue) or USPL1 (green) knock-down conditions. (**j**) Validation of differential exon usage of POL2RA detected by DEXseq through qRT–PCR, using primers briefly detailed in **h**, in HeLa cells treated with siControl (red), siTCAB1 (blue) or siUSPL1 (green). *n*=2, error bars represent s.e.m.

To assess whether RNA processing is perturbed after CB disassembly, we examined splicing patterns in the total RNA-seq data. Using an exon-centric approach (DEXSeq^45^), we identified 40 genes with differential exon expression in knock-down samples (Supplementary Data 4). The most enriched gene ontology category in this gene set was chromatin organization. 50% of these differentially expressed exons reside within 50 kb of 4C contact regions. Next, we performed a junction level analysis with a comprehensive classification of pairwise splicing events (Supplementary Data 5). A genome-wide summary of the RNA-seq and small RNA-seq data is presented in Supplementary Fig. 5. We identified 1,050 differentially spliced events (*q*-value<0.01, Fig. 6 a–c). The effects in the two knockdowns were generally in the same direction (*r*=0.66, Fig. 6c), supporting that TCAB1 and USPL1 are operating in similar pathways. We performed a meta-analysis of these two approaches with stringent criteria, and identified 85 genes with high confidence showing differences in their splicing (Supplementary Data 5). We found that a substantial proportion (42%) of these genes reside within 50 kb of 4C contact regions. Among the events differentially detected is an exon skipping event in *THOC5*, encoding a protein associated with the spliceosome. For another example, *BUB3*, involved in cell-cycle progression, shows a minor alternative splicing acceptor site used less frequently after TCAB1 or USPL1 knockdown (Fig. 6d,e).

Since splice junction analysis relies on gene annotations that are incomplete, we examined whether unannotated splice junctions are present in each sample, after adjusting for sequencing depth and transcription. These splice junctions may represent aberrant splicing events resulting from a loss of splicing fidelity. There were 11,515 unannotated junctions uniquely detected in siControl samples, indicating an inherent level of splicing noise in HeLa cells. Interestingly, we detected 30,132 unannotated splice junctions in siTCAB1 or siUSPL1 samples that were absent in siControl samples (Supplementary Data 5, Fig. 6f). The apparent lack of splicing fidelity was also observed at the gene level. These junctions belong to 9,077 genes, among which 6,424 (71%) had more than one unannotated junction. For example, five unannotated junctions for *SNRPE* were detected in the CB-disrupted condition but undetectable in the control HeLa. Fifteen genes with complex annotated splicing each had at least 20 unannotated junctions. Among these, the number of unannotated junctions within *ADARB1*, increased from 8 in the control to 31 in CB-disrupted cells. This gene encodes a protein responsible for adenosine-to-inosine RNA editing^46^, which raises the possibility of another layer of post-transcriptional regulation influenced by CBs. Furthermore, the majority of genes within CB contact regions had at least one unannotated junction after CB disassembly (72 and 83% of genes within 25kb of the *RNU1* and *H3F3A* 4C regions, respectively). However, the enrichment was not statistically significant when we considered genes of comparable expression status within the 4C-negative regions (70%), indicating that the altered splicing pattern reaches beyond the CB-proximal genes. There was minimal enrichment of small U RNAs encoded in the introns of abundant host genes which were differentially spliced after CB disassembly compared with all genes (4.1% versus 9%, *P*=0.025, Fisher's Exact Test) but this was not replicated in our unannotated splicing analysis (1% of junctions coincide with a small RNA gene). Thus, the broad post-disassembly of CBs increase of aberrant junctions suggests that CBs promote splicing fidelity, as well as efficiency of splicing.

We also observed additional effects that could not be directly attributed to splicing. The largest subunit of RNA polymerase (*POLR2A*) contains an exon differentially expressed in siTCAB1 samples. Notably, this is the last exon of the gene encoding the CTD tail, which is critical for the polymerase function and facilitates capping and splicing^47^ (Fig. 6h,i). Due to its location within the gene body, alternative polyadenylation is the most likely underlying mechanism. We confirmed these RNA-seq findings by qRT–PCR, including decreased expression of the Pol II CTD tail, as well as for several other genes (Fig. 6g,j). The effect on Pol II CTD suggests the possibility that some of the transcriptional changes in CB-disrupted cells observed away from *RNU1* contact regions may arise indirectly from the altered form and decreased level of RNA polymerase subunits.

The above phenomena regarding mRNA processing after CB disassembly are consistent with the highly reduced expression of many spliceosomal U RNA genes in CB-disrupted cells observed from the small RNA-seq data. Alternatively, reduced expression of histone genes has also recently been linked to decreased co-transcriptional splicing and increased splicing noise.^48^ We note that the splicing noise was elevated after CB disruption using two independent methods of disassembling CBs, through TCAB1 and USPL1.

## Discussion

CB formation is thought to involve stochastic self-organization, initiated by nucleating factors coalescing and accumulating at distinct transcriptionally active nuclear sites. We have previously documented that CBs and physically associated HLBs can be formed *de novo* by tethering unprocessed histone pre-mRNAs or specific CB components on a gene locus which then act as seeding elements^12^,^49^. Live-cell imaging studies have shown that CBs can go through fusion and fission process as the cell cycle progresses^50^. Highly active transcription may also promote the specific gene locus association with CBs, as evidenced by an engineered inducible U2 snRNA gene array targeting a CB in proximity by an actin-dependent long-range movement after transcriptional activation^37^. Our current study shows that small U RNA genes of all categories across the genome are capable of forming specific intra- and inter-chromosomal gene clusters around CBs, consistent with a recent coilin ChIP-seq study that identified several U RNA gene loci as potential coilin-interacting sites^51^. Since the early gene interactions presumably occur stochastically before the formation of structurally stable CBs, the highly active tandemly repeated gene arrays such as *RNU1*, *RNU2*, and histone gene clusters may eventually coalesce with other transcriptionally active sn/snoRNA genes on other chromosomes around CBs. Our RNA-seq analysis also revealed an additional layer of self-regulation for CB maintenance. Disruption of CBs by specific knockdown of component factors invoked a feedback control mechanism to compensate for the loss of CB structure, including upregulation of critical CB components (coilin and Gemin5) and downregulation of a CB-interacting factor of the Integrator complex (*INTS3*) which regulates pre-U RNA 3'-end processing^43^,^44^. Further work will be necessary to understand the selectivity of such regulation.

Multiple complementary approaches were used in our study to demonstrate that chromosome 1 may play a potentially indispensable role in the nucleation of CBs. Chromosome 1 adopts a topological arrangement around the intra-chromosomally positioned CB, frequently juxtaposing the two opposite chromosomal ends in close proximity. The physical proximity of a vast majority of highly expressed small U RNAs and histone genes on chromosome 1 to the CB may create a spatial configuration that accelerates the processing of accumulated or stalled nascent transcripts from these loci.

Since CBs are often physically associated with HLBs^12^,^29^, we analysed their relationships and were able to distinguish which specific histone gene clusters contribute to their formation. Our six-colour DNA FISH revealed that the large major histone gene cluster *HIST1* on chromosome 6 primarily nucleates CB-protruding HLBs, and the smaller minor histone gene cluster *HIST2* on chromosome 1 is mostly associated with the CB and a smaller HLB. These data support the distinct roles of different histone gene clusters on the two chromosomes.

Through a combination of 4C-seq, total RNA-seq, small RNA-seq and qRT–PCR analysis, we characterized the genome-wide impact of CBs on chromosome architecture and CB-dependent gene regulatory events. In contrast to the idea of NBs as separate physical entities from the nuclear genome, our findings highlight the tight interaction between CBs and select regions of the genome and the consequent influence of CBs on genome organization. CBs promote a genome conformation that supports co-localization of sn/snoRNA genes distributed throughout the genome, and sustain the highly abundant expression of sn/snoRNA and histone genes. We find that CBs exert a greater regulatory effect on moderately expressed U RNAs, as small U RNAs highly expressed in tissue-matching primary controls were relatively unaffected by CB disassembly. Although the expression of a few spliceosomal small U RNAs has been linked to CB formation^36^, our study is the first to demonstrate CB-regulated genome-wide effects on the expression profile of sn/snoRNAs and histone genes. In addition to the splicing-related RNA genes, including all major and minor spliceosomal components, all other categories of sn/snoRNA genes are found to be in contact with CBs and exhibit CB-dependent expression.

In contrast, the global expression profile of protein-coding genes does not significantly depend on CBs. However, several genes related to efficient pre-mRNA splicing show CB-sensitive expression, and may warrant special consideration. Among these was *SMC1A* whose expression decreased in CB-disrupted cells. In addition to its prominent role in the cohesion complex, which encourages faithful sister chromatid pairing during mitosis, *SMC1A* has also been implicated in the DNA damage response^52^ and may be a potential spliceosome accessory factor^53^,^54^. *SMC1A* has also been identified as a mediator of local promoter–enhancer element interactions, as well as chromatin looping^55^,^56^. Thus, any misregulation of this gene may entail disproportionate effects on the genome architecture and integrity, gene expression, and splicing. Consistent with previous reports on the co-operative relationship between CBs and spliceosomal core Sm protein expression,^57^ we also note CB-dependent expression of other genes directly related to the spliceosome, including *SNRPE* (an integral member of the heptameric Sm-ring complex required for the stability of snRNPs), *PRPF3* and *SNRNP27* (components of U4/U6.U5 tri-snRNP complex and recycled after each round of RNA splicing), *SNRPA1* (U2 snRNP-specific A' protein), and splicing inhibitors such as variant hnRNP H. CBs may also influence DNA and RNA metabolism through the regulation of mRNA synthesis and trafficking (*MECP2, MBD1, PEBPC1, THOC5, RPAIN*), rRNA and tRNA production (*RPL4, POLR1E, EIF6, DUS3L*) and DNA damage repair (*POLM, SMC1A*).

We present evidence for the regulation of splicing fidelity by CBs. Exon and junction analyses of total RNA-seq data show that the noise in the error-prone splicing process increases further when CBs are disturbed. Consistent with the perturbed expression of spliceosomal components, we observe a substantial usage of alternative exons following CB disassembly, from genes within regions proximal to CBs. In addition, we noted an increased number of aberrant splicing events indicative of errors in splicing. Although junction detection and comparative analysis are computationally challenging, we used relatively deep sequencing, multiple splicing analysis algorithms, and two independent methods of CB disassembly and found these results suggesting an elevated level of splicing noise in CB-disrupted cells.

Several possible scenarios can be envisioned to link CB function to RNA splicing fidelity. First, loss of CBs and decrease in expression of spliceosomal RNAs result in improper production of spliceosomal complexes. This is unlikely to be the sole contributing factor in this system as spliceosomal snRNPs display long half-lives and is in vast excess^58^,^59^. Second, improper processing of intron-encoded snoRNAs results in aberrant splice site selection. We found no significant enrichment of intron-encoded U RNAs in those host genes which contain unannotated splicing patterns after CB disassembly compared with genomic background. Third, CB-dependent rescue of snoRNAs (scaRNAs in particular) from a degradation pathway is lost, resulting in insufficient post-transcriptional base modification of the essential U RNA components of the major and minor spliceosomal pathways. This has previously been linked to changes in splicing fidelity^60^,^61^ and, in combination with the decreases in U RNA gene expression described in Fig. 1, may explain the changes in splicing we observe following CB disassembly. Finally, a recent study has indicated that decreases in histone levels, as we report here, may lower co-transcriptional splicing, increase intron retention and induce changes in alternative splicing dynamics^48^. Thus, through HLB and histone gene regulation, CBs may influence RNA maturation.

There may be further downstream genome-wide effects of CBs beyond the phenotypes detected immediately after CB disassembly in the current study. It is noteworthy that some key regulators of the DNA/RNA metabolism are among the small number of genes with CB-dependent expression (Supplementary Fig. 6). Perhaps a direct regulation of the chromatin landscape may arise through CB's influence over histone gene cluster organization and expression. Interestingly, CBs also support the expression of *SNRPE*, an essential component of the Sm ring on U7 snRNP used for histone pre-mRNA processing^62^. Other potential mechanisms that may propagate broadly include poly(A) tail selection, mRNA stability and export, as hinted by altered genes such as *THOC5* and *PEBPC1*. For example, aberrant splicing of *THOC5* can have an impact on a large number of genes, since it influences the export and expression of the vast majority of immediate early genes induced by extracellular stimuli. Differential exon usage in the CTD tail of the largest subunit of RNA polymerase II (*POLR2A*) could also have wide-ranging genomic effects. Taken together, there may be broad longer-term consequences of CB disassembly beyond the selective changes in gene expression that were observed immediately after CBs are perturbed.

**Figure 7:**
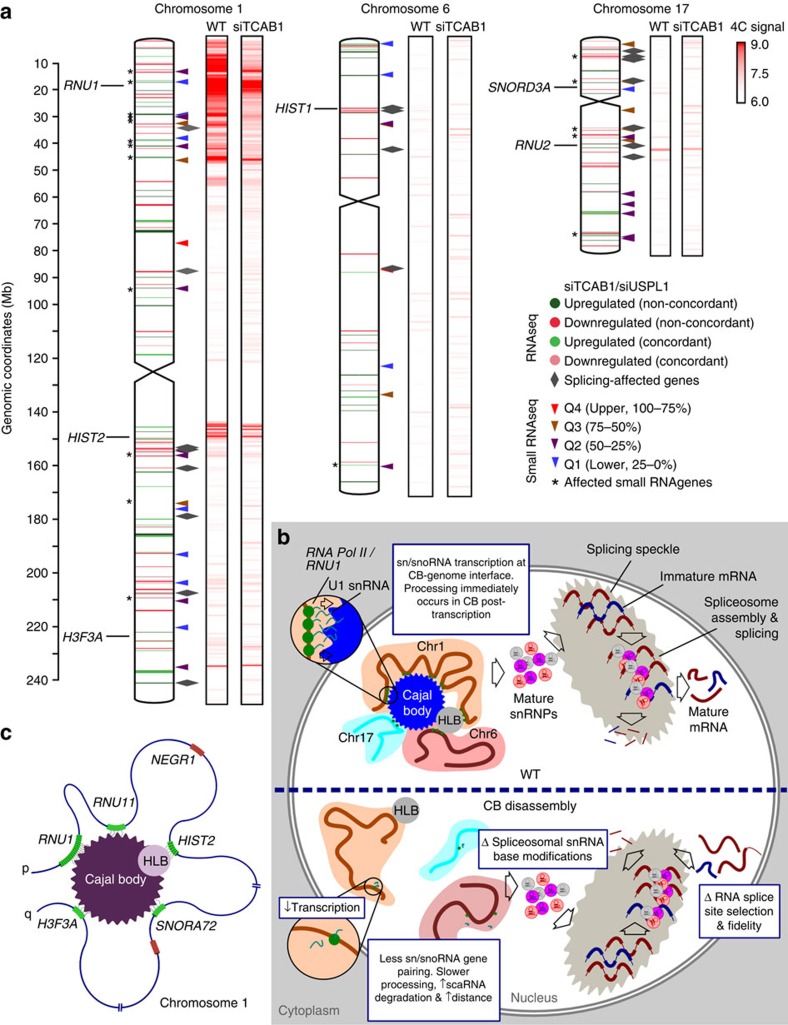
Cajal bodies orchestrate genome-wide clustering of spliceosomal and histone-related genes. (**a**) Composite image detailing inter- and intra-chromosomal contact 4C interactions, gene expression changes and alternative splicing events following CB disassembly (siTCAB1) in HeLa cells for chromosomes 1, 6 and 17. The locations of gene loci of interest (*RNU1*, *RNU2, SNORD3A*, as well as *HIST1, HIST2* and *H3F3A*) are shown. Upregulated genes (RNA-seq) are denoted by green chromosome bands (non-concordant=dark green, concordant=light green) and downregulated by red (non-concordant=dark red, concordant=light red). Baseline (siControl) expression of small RNA U genes are denoted by triangles (red=Q4 (upper, 75–100%), brown=Q3 (50–75%), purple=Q2 (25–50%) and blue=Q1 (lower, 0–25%)) Grey triangles denote the alternative splicing events, *=expression changes of genes (small RNA-seq). 4C-seq physical contact profile is shown in red (4C signal, log10 scale). (**b**) Proposed role of CBs (blue) in genomic organization and maintenance of tightly regulated splicing processes in HeLa cells. (**c**) Schematic representation of a proposed topological rearrangement of chromosome 1 by a CB (purple) and physically associated HLB (grey) resulting in regulation of gene positioning. CB- or HLB-interacting gene loci are shown in green, whereas non-interacting regions are shown in red.

On the basis of the regulation of gene expression and splicing fidelity, we propose a previously unappreciated role of CBs as genome-organizing centres, in addition to its established function in snRNP biogenesis and recycling pathways. Our data also establish that highly expressed, frequently CB-proximal small U RNA gene loci, such as the highly expressed *RNU1* or *RNU2* gene arrays (Fig. 7a), probably serve as key CB nucleation sites capable of targeting all the other categories of small RNA genes. Once structurally formed, CBs promote the looping and aggregation of a limited cohort of sn/snoRNA and histone (via the physically associated HLBs) genes in their proximity, particularly within chromosome 1, to regulate their expression (Fig. 7b,c)^37^. In the absence of CBs, these topological repositioning events do not occur, resulting in downregulation of small U RNAs and changes in genome-wide chromosomal interactions. Depletion of spliceosomal U RNA genes and scaRNAs that guide their base modifications is likely to alter RNA splicing fidelity, as previously described^63^. Our study opens the way for further investigations of the longitudinal effects of CB formation and disruption in a controlled system.

## Methods

### Cell culture

HeLa human cervical carcinoma cells were maintained according to standard procedures in DMEM containing 10% (v/v) fetal bovine serum, glucose (4.5 g l^−1^), and 5% CO_2_. The primary human cervical epithelial cell line (Epi) was purchased from Cell Applications and maintained in propriety Epi growth medium/5% CO_2_. D551 primary skin fibroblasts were kindly provided by Dr Dominik Duelli and cultured under the same conditions as HeLa cells. One day before lysis, Epi cells were transferred to DMEM and maintained under the same conditions as HeLa and D551 cells to account for differences arising from culture conditions.

### siRNA oligonucleotide transfection and cell cycle arrest

Delivery of siRNA into HeLa cells was achieved using DharmaFECT 1 transfection reagent (Dharmacon) according to manufacturer's instructions. TCAB1 siRNA was acquired from Qiagen (SI00388941), USPL1 siRNA (sc-76875) and non-targeting control siRNA (sc-37007) from Santa Cruz. Briefly, 100 nM siRNA was transfected on two consecutive days and cells were collected 24 h after the second round of transfection.

To induce an artificial cell cycle block at the G1/S-phase boundary, HeLa cells were treated with a final concentration of 5 mM thymidine for 18 h. Cells were released and grown in fresh media for 9 h, followed by a second block with 5 mM thymidine for 18 h. Cells were immediately collected for analysis after the second thymidine block without release into fresh media.

### Hybridization probes

Probes were purchased from BACPAC, tested by PCR, metaphase spreads and co-staining with CTs in primary diploid cells. The DNA hybridization probes were generated using specific BACs by nick translation with 488-dUTP from Abbott Laboratories, 552-dUTP and Cy5-dUTP from GE Healthcare, and 594-dUTP and 680-dUTP from Biotium.

### DNA FISH

Cells grown on glass 12 mm circle coverslips were washed with PBS and fixed with 4% PFA in PBS for 10 min at room temperature. After rinsing in PBS, cells were permeabilized with 0.5% Triton X-100 in PBS for 20 min on ice and were washed with PBS. Subsequently, the cells were incubated in 0.1 N HCl for 15 min, and then washed twice in 2 × SSC for 10 min, before equilibration in 50% formamide/2 × SSC for 30 min. For one 12-mm round coverslip, ∼150 ng of DNA FISH probe of each fluorescent dye were combined together and precipitated with 3 μg of Cot-1 DNA (Roche) and 1 μg of yeast tRNA in ice-cold absolute ethanol by sodium acetate, spun down, dried and resuspended in 7 μl of hybridization solution (10% dextran sulfate/50% formamide (pH 7.0)/2 × SSC/1% Tween20). The cocktail was denatured at 85 °C for 5 min, put briefly on ice, incubated with cells at 85 °C for 5 min, sealed with rubber cement and hybridized in a humidified chamber overnight at 37 °C. After hybridization, cells were washed three times in 50% formamide/2 × SSC for 5 min at 45 °C, washed three times in 1 × SSC for 5 min at 60 °C and in PBS for 5 min. Then cells were incubated with the rabbit antibody against coilin (Santa Cruz) for 1 h, washed three times in PBS. Cells were then incubated with donkey anti–rabbit secondary antibody conjugated with 405S dye (Jackson ImmunoResearch Laboratories) for 1 h, washed three times in PBS for 5 min, and mounted using ProLong antifade mounting reagent without DAPI (Life Technologies). Cells were observed on Zeiss LSM710 or LSM780 confocal microscope using a 63 × 1.40 NA objective using z-sectioning (z-250 nm) with spectral imaging and linear unmixing using Zeiss Zen software.

### 3D DNA chromosome painting

The protocol is identical to DNA FISH procedure except chromosome painting probes were hybridized with fixed cells for three nights at 37 °C. Theoretical association frequencies were assessed after quantification of the nuclear and CT1 areas using ImageJ. The radii of these areas were then estimated and used with the following formula:


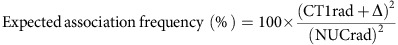


CT1rad=average radius of chromosome 1, NUCrad=average nuclear radius, Δ=threshold for edge–edge distance between CB and Chr1.

### Chromosome conformation capture followed by sequencing

The 4C assay was performed as previously described^64^. Briefly, cultured HeLa cells were cross-linked in 2% formaldehyde at 37 °C for 10 min. After being quenched with glycine, cells were lysed using 10 mM Tris-HCl, pH8.0, 10 mM NaCl, 0.2% NP-40) supplemented with 1 × complete protease inhibitors (Sigma) at 4 °C for 1 h. Cross-linked chromatin was digested overnight with an excess of *Hin*dIII enzyme (New England Biolabs), and then DNA ends were ligated under diluted conditions that favour junctions between cross-linked DNA fragments. NlaIII (New England Biolabs) was used in a second round of digestion, and DNA was re-ligated. Primers were used to PCR-amplify 4C DNA can be found in Supplementary Data 1. Each experiment was independently performed twice. 4C DNA samples were barcoded and sequenced on Illumina HiSeq2000 according to the Illumina paired-end 100 bp sequencing protocol.

4C-seq data were analysed by the following procedure: Raw data were initially processed by de-multiplexing, bait-trimming and quality filtering. Reads were trimmed to the first 50 nucleotides before mapping them onto hg19 using Bowtie. A primary 4C profile was generated by counting the number of reads overlapping each non-overlapping genomic bin of size 10 kb. For smoothed 4C profiles, the running 100 kb window-average of the bin counts was assigned to the centre of each genomic 10 kb bin. After confirming profile reproducibility, duplicate 4C files were pooled by combining reads from two biological replicates for each condition. The smoothed 4C profiles were generated from the pooled data, and 0.1 was added before converting to the log2 scale.

Enrichment of small RNA and histone genes within 10 kb of 4C contacts was assessed by generating 100 random samples of matching size and calculating an empirical *P* value. For 4C contact regions, we retrieved the coordinates of the smoothed profile with the above-defined log2 4C signal >7.

### RNA preparation and qRT–PCR

Total cellular RNA was extracted with RNeasy Mini kit (Qiagen) in accordance with the manufacturer's instructions, before sequencing. For quantitative RT-PCR, cDNA was synthesized using the iScript cDNA Synthesis kit (Bio-Rad). Real-time PCR was performed in triplicate using the iQ SYBR Green PCR Supermix (Bio-Rad). The abundances of the mRNAs of interest in each sample were normalized to that of β-actin mRNA, and fold changes in target mRNAs relative to their basal abundances were calculated by the 2^−ΔΔCt^ method. Each experiment was performed in two independent repeats.

### Small RNA sample preparation and sequencing

Ribosomal RNAs (rRNA) were removed from total RNA samples using Ribo-Zero rRNA Removal Kits (Epicentre). The total RNA samples (after depletion of rRNA) were size-fractionated on a 6% TBE-Urea polyacrylamide gel to enrich for molecules in the range of 50–400 nt. Small RNA libraries and sequencing for each sample were performed by LC Sciences (Houston, TX). In brief, RNA fragments were dephosphorylated and ligated with 3′-RNA adaptor. The resulting ligated RNA fragments were reversed-transcribed and ligated with 5′-DNA/DNA adaptor and PCR amplified (15 cycles) using primers on both adaptors. Subsequently, PCR products were purified and small RNA library was sequenced on Illumina GAIIx (Illumina, Santa Clara, CA) for single-end 35-mers or on Illumina HiSeq for single-end 51-mers following the manufacturer's protocol. Raw sequence FASTQ files were generated by using Illumina's software pipeline.

### Small RNA-seq data analysis

Raw reads were adaptor-trimmed and filtered to remove low-quality reads. We downloaded the hg19 coordinates for all the annotated snRNAs from the UCSC browser based on ENSEMBLE version 70 (January 2013) and a query for RNU, snoRNA, snoRD and scaRNA. This resulted in 1,649 non-redundant small RNAs and their coordinates. In all, 35-mers were mapped to the hg19 reference genome using Bowtie 1.0.0 with the parameters ‘-p 4 -v 1 -m 1'. We computed fragments per kilobase per million mapped reads (FPKM) using Cufflinks over the annotated small RNAs using the downloaded coordinates. We also verified that the numbers of reads mapping to mRNAs were negligible as expected from the size selection. After confirming reproducibility of global profiles in biological replicates, the average of the duplicate log2 FPKM values was used to quantify small RNA expression. Expression in a knock-down sample was compared with expression in untreated HeLa (control 1) and in siControl-treated HeLa (control 2). An increase/decrease in expression was deemed significant if it is relative to both control 1 and control 2.

### RNA-seq sequencing and genome alignment

Libraries were generated with the Illumina Truseq protocol (Illumina, San Diego, CA) and TruSeq sample prep kits (catalogue ID: FC-122-1001). Libraries were sequenced on a HiSeq2000 instrument (Illumina) for 2 × 101 cycles, generating 101 base-paired-end reads. All samples were run on the same flowcell, multiplexed with 4 samples per lane. The Illumina basecalling and read generation algorithm versions were RTA 1.12.4.2 and CASAVA-1.8.2. Sequencing, read pre-processing and alignment were performed by the Center for Cancer Research (CCR) Genomics Core (Frederick, MD). Raw reads were trimmed before alignment. The trimming software used was ea-utils FasqMcf software (http://code.google.com/p/ea-utils/wiki FastqMcf Expression Analysis, Durham, NC). The trimming parameters used were: -l 15 -q 0 -u -P 33 (minimum retained sequence length=15, quality threshold causing base removal=0, enable Illumina PF filtering, Phred 33 scale). Trimmed reads were mapped to the hg19 reference genome with TopHat v.2.0.8 (ref. 65, accepting only unique alignments (parameters -g 1 -r 10 --mate-std-dev 100), with the Bowtie2 alignment engine^66^. Gene model annotation for hg19 from Ensembl was provided to the aligner with the -G parameter.

### List of contact genes from 4C data

For each 4C experiment (*RNU1* and *H3F3A*), lists of putative interacting genes were obtained as follows: from single-base positions, BED files of extended ranges (±10 kb, 25 kb and 50 kb) were generated. A BED file of extended gene regions (start to end) was generated from UCSC annotation and the R package ‘rtracklayer' and ‘GenomicFeatures'. We performed intersections of these BED files with bedTools^67^, requiring that the midpoint of the extended gene overlap with the 4C region (intersectBed –f 0.50). From overlapping genes, we applied a gene expression criterion to define putative 4C-positive interactors (gene expression in wild type in the top 5% of all genes). A control set (4C-negative) was defined as genes in the top 5% of gene expression in wild type, but residing >50 kb from 4C contact regions. Lists are included in Supplementary Data 1.

### RNA-seq data analysis

All expression analysis was performed against the UCSC hg19 genes.gtf annotation, downloaded from the iGenomes collection (Illumina). Gene-level expression in units of FPKM were generated with Cufflinks v.2.1.1 (ref. 68, using default parameters except for turning off suppression of rare isoforms (-F 0). Differential expression was performed using Cuffdiff v.2.1.1, using ‘quartile' as the library normalization method. Gene expression was also quantified using the HTSeq program v.0.5.4p1 (htseq-count parameters -q -m union -s no -i gene_id -t exon). Count results were analysed with DEseq v.1.16 (ref. 45). We used these results to generate a high-confidence set of gene expression calls based on concordance between the two methods (Supplementary Data 3). Complete gene-level output from each program is provided in Supplementary Data 3.

Splicing was analysed by multiple complementary approaches. We used the Splicing Analysis Toolkit (Spanki) v.0.4.3 (ref. 69) to quantify splice junction coverage from alignment files, and to quantify proportion spliced in for splicing events. The AStalavista tool^70^ was used to characterize events from annotated transcript models. This tool requires annotation in a GTF format only available for annotation distributed by Ensembl. We downloaded the most recent Ensembl annotation (Homo_sapiens.GRCh37.75.gtf), and identified 638,467 pairwise splicing events with AStalavista v.3.2. The Spanki toolkit was also used to assign unannotated splice junctions to genes. Splicing was also quantified using the Jensen–Shannon Divergence metric from the Cuffdiff program^68^. To analyse unannotated splice junctions unique to knockdown, we generated two metrics to normalize junction detection based on transcription and sequencing depth.

Count-based analysis within exons was performed with the DEXSeq package^71^, which quantifies exon expression normalized for transcription and sequencing depth (KD1_reads_per_million=coverage in KD1/millions of reads, Wt_Expect_KD1_depth=millions of reads * KD1_reads_per million, KD1_reads_per_FPKM=coverage in KD1/FPKM in KD1, Wt_Expect_KD1_FPKM=FPKM * KD1_reads_per_FPKM). Gene ontology functional enrichment analysis was performed with the DAVID tool^72^ and the GOStats tool^73^.

### High-throughput microscopy

Following DNA FISH/IF staining as described above, except for seeding of cells in 384-well clear-bottom plates (CellCarrier, PerkinElmer), cells were imaged using an Opera QEHS (PerkinElmer) spinning—disk confocal high-throughput imaging system. Images were acquired in three sequential exposures in four channels:

(i) DAPI: excitation laser 405 nm, emission filter: BP 450/50, exposure 1.

(ii) *RNU1*-488 nm: excitation laser 488 nm, emission filter: BP 520/35, exposure 3.

(iii) Variable gene of interest (VGOI) BAC-568nm: excitation laser 561 nm, emission filter: BP 600/40, exposure 2.

(iv) Cy5: excitation laser 640 nm, emission filter: BP 600/40, exposure 1.

All channels were acquired using a 405/488/561/640 primary excitation dichroic mirror and a 568 detection dichroic mirror. The images were acquired using a × 40 water objective lens (NA 0.9, focal depth 1.2 μm) with 6 optical steps of 1.0 μm, with a camera binning of 2. The pixel size was 320 nm.

### Automated image analysis

Images were analysed with the Acapella 2.0 or 2.6 software (PerkinElmer). To quantify distances between FISH signals and coilin, we modified a custom image analysis script in Acapella for nuclear segmentation and spot detection^74^. In brief, images from different z-planes and from the same channel and field of view were maximally projected in 2D. Nuclei were segmented using the maximally projected images in the DAPI channel. Nuclei touching the image border were not considered for further analysis. The segmented nuclear region was then used by an intensity-based spot detection algorithm to segment the FISH and IF signals in the maximally projected RNU1-488 nm and VGOI-568nm channels and the Coilin bodies were detected using the maximally projected Cy5 channel. For every FISH and IF signal, its brightest pixel was identified as the spot centre. All the distances between spots of different colours were measured by calculating the Euclidean distance between their spot centres using their respective *x*- and *y*-axis pixel coordinates in the image. All the single-cell and single-distance data was indexed on a per-well, per-imaging field, per-cell and per-spot basis for further downstream analysis. Single-object data (Cell- and distance-level) was exported as ‘.txt files'. The Acapella 2.0 or 2.6 image analysis scripts and analysis parameters, as well as all microscopy images generated during this project, are available on request to the authors.

### Single-distance data analysis

Data analysis on single-object level data was performed using the R statistical analysis software^75^. In brief, only spot distances from cells with at least 1 FISH signal per gene and 1 CB were considered for further analysis. Minimum *RNU1*-gene of interest and *RNU1*-CB were calculated on a per *RNU1* FISH spot basis. CB-gene of interest distances were calculated on a per-CB spot basis. Since all the distances were indexed based on their identity of the spots they were calculated from, it was possible to assign a minimum CB-gene of interest basis distance for a particular *RNU1* FISH Spot based on its minimum *RNU1* FISH Spot/CB minimum distance. A CB-independent *RNU1*-gene of interest interaction was defined as a *RNU1* FISH Spot having a minimum *RNU1*/gene of interest distance of ≤0.96 μm (3 pixels), and a minimum *RNU1*/CB distance of >1.6 μm (5 pixels, threshold distances were selected following visual scoring of images before automated analysis, Supplementary Fig. 4d,e). A CB-dependent *RNU1*-Gene of Interest interaction was defined as a *RNU1* FISH Spot having a minimum *RNU1*/gene of interest distance of ≤0.96 μm (3 pixels), and a minimum *RNU1/*CB distance of ≤1.6 μm (5 pixels). A CB bridging event was defined as a *RNU1* FISH Spot having a minimum *RNU1*/CB distance of ≤1.6 μm (5 pixels), and the respective minimum CB/VGOI distance of ≤1.6 μm (5 pixels). Interaction frequencies between each of the genes and *RNU1* were calculated by computing the percentage of cells carrying at least one event as defined by the criteria listed above. The ‘.rmd' file containing the R script using for the single-distance data analysis is available on request.

## Additional information

**How to cite this article**: Wang, Q. *et al.* Cajal bodies are linked to genome conformation. *Nat. Commun.* 7:10966 doi: 10.1038/ncomms10966 (2016).

## Supplementary Material

Supplementary Information Supplementary Figures 1-6, Supplementary Table 1 and Supplementary References

Supplementary Data 1 Supplementary Data 1 4C-seq analysis following TCAB siRNA treatment, including a list of genes which are within 10kb, 25kb or 50kb of 4C contact regions using *RNU1* or *H3F3A* bait primers.

Supplementary Data 2 Small RNA-seq analysis of diploid cervical epithelial cells, D551 diploid fibroblast cells, HeLa siControl, HeLa siTCAB1 and HeLa siUSPL1 samples. Analysis includes RNA expression levels and intersection with 4C contact regions.

Supplementary Data 3 Differential gene expression analysis (using DEseq/CuffDiff) and Gene Ontology analysis of total RNA-seq datasets from HeLa siControl, HeLa siTCAB1 and HeLa siUSPL1 samples.

Supplementary Data 4 Differential splicing analysis of RNA-seq datasets using DEXseq/Spanki and subsequent Gene Ontology analysis.

Supplementary Data 5 Unannotated splice junction analysis following CB disassembly using TCAB1 and USPL1 siRNAs.
