# Supplementary material for: Cajal bodies are linked to genome conformation
Source: Nat Commun. 2016 Mar 21;7:10966. doi: 10.1038/ncomms10966 (PMC4802181; doi:10.1038/ncomms10966)
Supplement: Supplementary Information — Supplementary Figures 1-6, Supplementary Table 1 and Supplementary References [file ncomms10966-s1.pdf]

Supplementary Figure 1

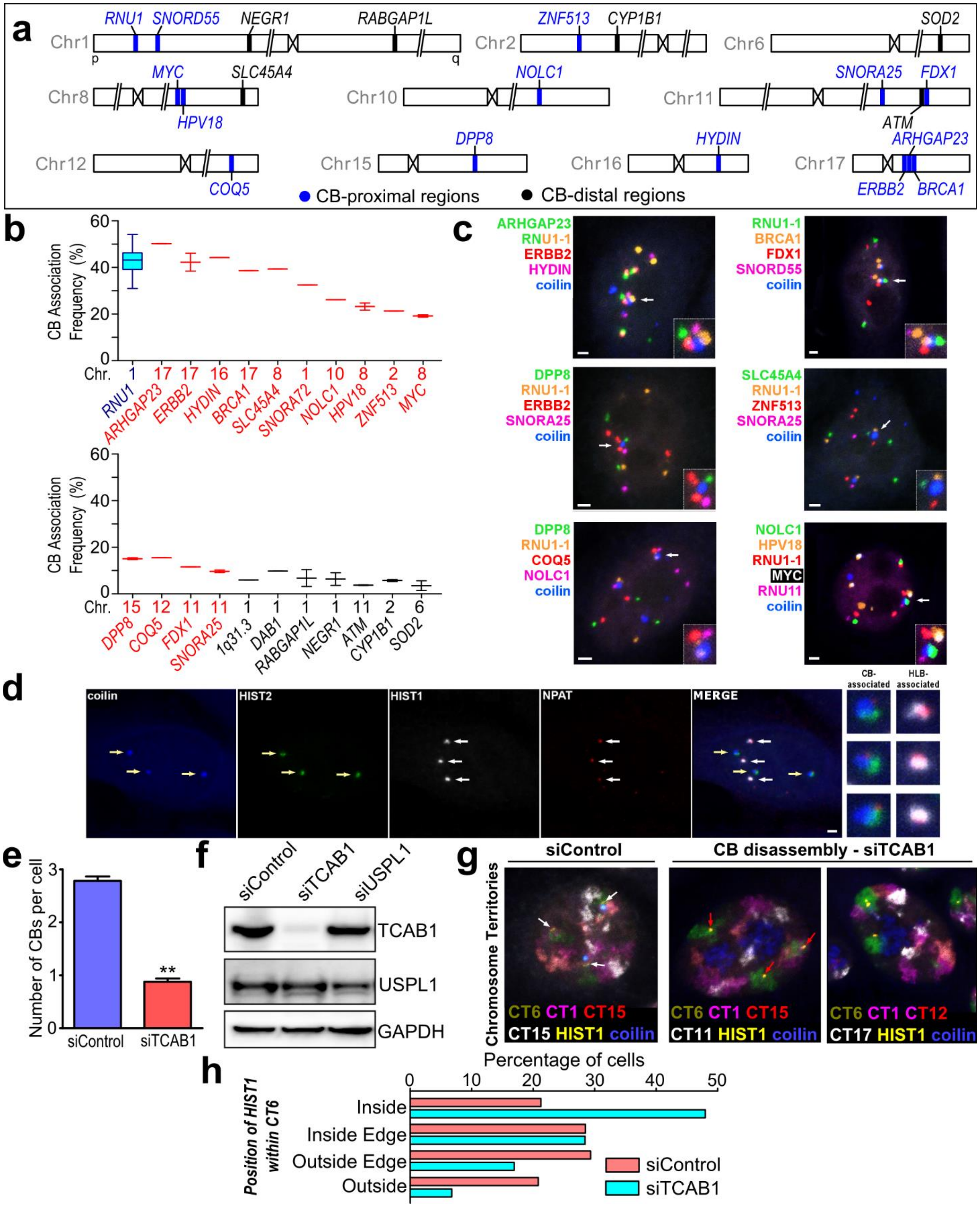

(a) Approximate chromosomal location of BAC DNA-FISH probe target sequences used in this study. Blue text denotes CB interacting (4C contact positive) gene loci and black denotes non-CB interacting (4C contact negative gene) loci, 6-color fluorescent microscopy analysis of four chromosome territories

(CTs), CB interacting gene locus (*HIST1*) detected by DNA-FISH and CB localization visualized by CB marker protein coilin in aneuploid HeLa (CB-positive) and CB-deficient (siTCAB1) cells, (b) Box and whisker plot of DNA-FISH loci-CB association frequencies determined by manual scoring DNA FISH foci proximity to CBs using 6-color DNA FISH fluorescent microscopy images. Chromosome location is indicated below the X-axis. Black = non-CB interacting gene loci. Data represents assessment of at least 150 HeLa cells, (c) Validation of highly CB- interacting trans-chromosomal genomic regions identified by 4C analysis with the *RNU1* bait by using 6-color DNA FISH fluorescent microscopy analysis of CB-interacting loci (various) by DNA-FISH and coilin (blue) immunostaining in HeLa cells targeting the HLB marker protein NPAT (red) and CB marker coilin (blue), respectively, (d) Microscopic fluorescent analysis of the role of major histone locus (*HIST1*) on chromosome 6 and minor histone locus (*HIST2*) in the formation of CBs and HLBs in HeLa cells. *HIST1* (green) and *HIST2* (white) DNA FISH foci denote the major and minor histone gene cluster loci on chromosomes 6 and 1, respectively. Immunofluorescent detection of HLBs and CBs was achieved using specific antibodies, (e) Cajal body numbers in siControl and siTCAB1-treated HeLa cells. N=2 (400 cells), **=$p<0.01$, significance was assessed by Student's T-test. Error bars represent SEM, (f) Representative TCAB1 and USPL1 immunoblots using extracts from HeLa cells exposed to indicated siRNA for up to 72 hours. GAPDH was used as a loading control, (g) (h) Quantitative assessment of HIST1 localization in control HeLa cells (red) and CB-deficient cells (siTCAB1, blue). Scoring reflected the relative position within the CT6 territory, from completely internal (inside), to positions reflecting more active states either within or on the outside edge of the CT (inside edge & outside edge, respectively) as well as gene completely looped out of the CT (Outside).

Supplementary Figure 2

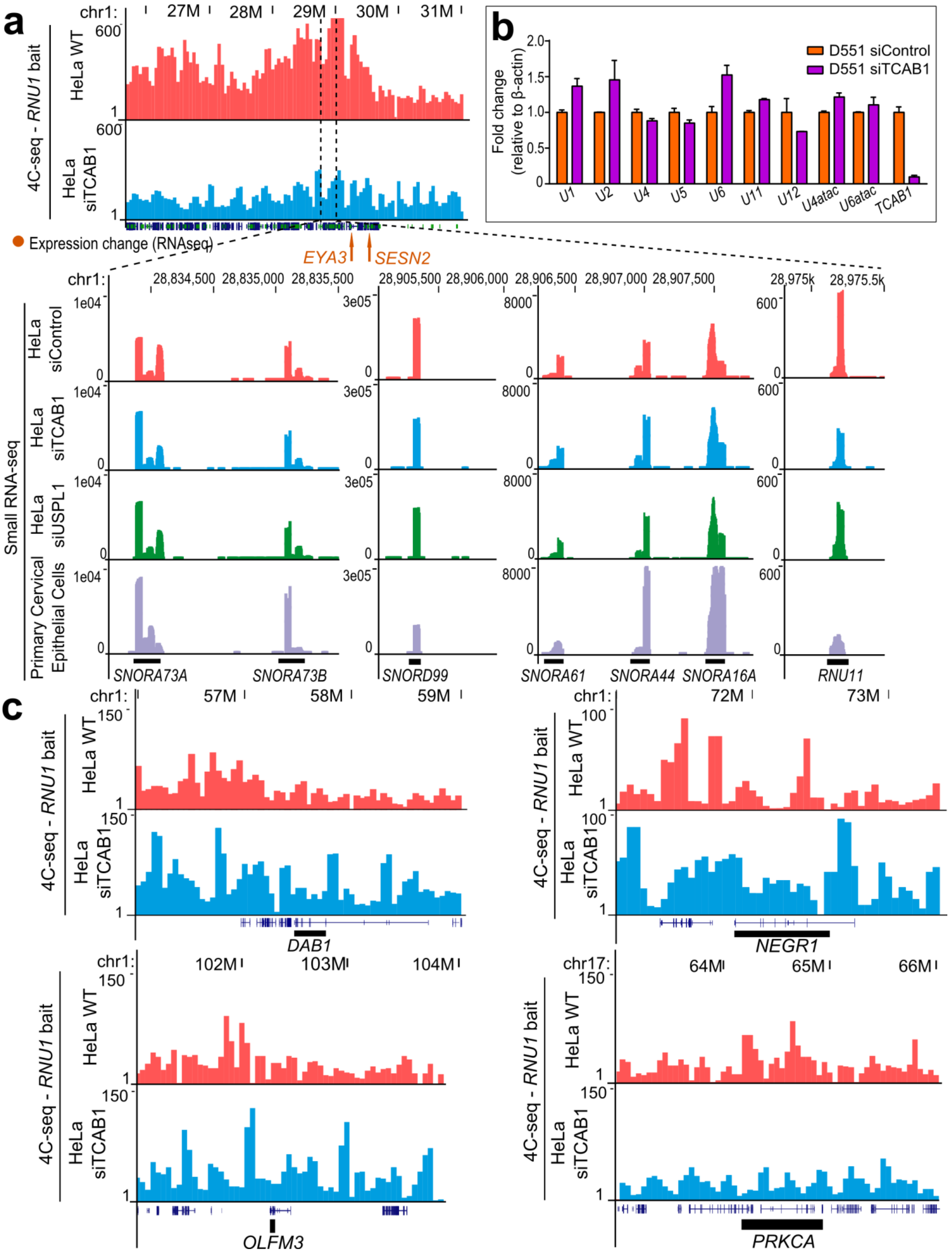

(a) Representative 4C-seq contact profile of intra-chromosomal, CB-dependent interactions using a *RNU1* bait sequence for HeLa WT (pink) and siTCAB1 (blue) treatments. A number of small U RNA genes were enriched within this region (dotted lines) which were analyzed following siTCAB1 and siUSPL1 (green) treatment by small RNA-seq. The expression of these genes within human primary cervical epithelial cells is also shown (purple). A number of small U genes were determined to be insensitive to TCAB1 or USPL1kd, possibly distinguished from sensitive genes by their higher expression in primary epithelial cervical cells. The Y-axis displays the read counts/genome fragment, (b) Expression of histone genes in D551 primary diploid fibroblasts following depletion of TCAB1 by siRNA. N=2, error bars represent SEM. D551 siControl = orange, D551 siTCAB1 = purple, (c) Representative 4C-seq contact profile validations of non-CB-interacting (4C negative contact) loci used in DNA-FISH experiments as negative control. Profiles display *RNU1* bait to negative 4C region contacts on chromosome 1 and 17 in control (pink) and siTCAB1-treated (blue) HeLa cells. The Y-axis displays the read counts/genome fragment.

Supplementary Figure 3

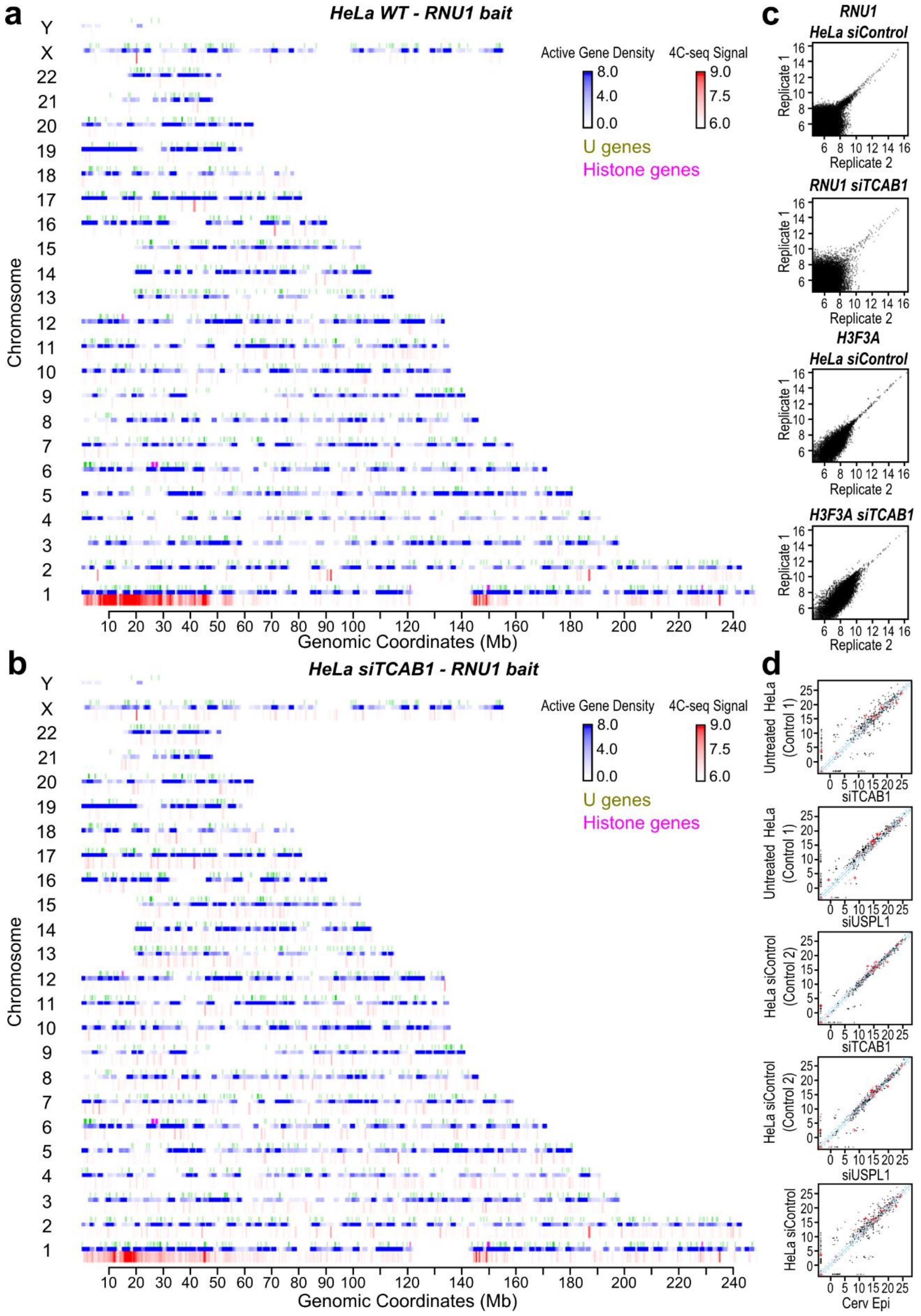

(a)(b) Genome-wide *RNU1* 4C-seq physical contact profile (red, log10 scale) in WT HeLa cells following CB disassembly using TCAB1 siRNA (B). For comparison, active gene density is shown

adjacently (blue, log10 scale), as well as small U RNA gene and histone gene expression (green and red, respectively), (c) 4C-seq replicate reproducibility for *RNU1* and *H3F3A* bait analyses in HeLa cells treated with either control or TCAB1 siRNA treatments (siControl or siTCAB1, respectively). Spearman's correlation coefficients: *RNU1* bait – HeLa siControl = 0.182; *RNU1* bait – HeLa siTCAB1 = 0.080; *H3F3A* bait – HeLa siControl = 0.870; *H3F3A* bait – HeLa siTCAB1 = 0.903, (d) Small RNA-seq analysis comparing HeLa cells treated with either control or TCAB1 siRNA treatments (siControl or siTCAB1, respectively) and primary cervical epithelial cells (Cerv Epi). Red and pink crosses indicate loci within 10kb and 50kb of *RNU1* 4C contact regions in WT HeLa, respectively.

Supplementary Figure 4

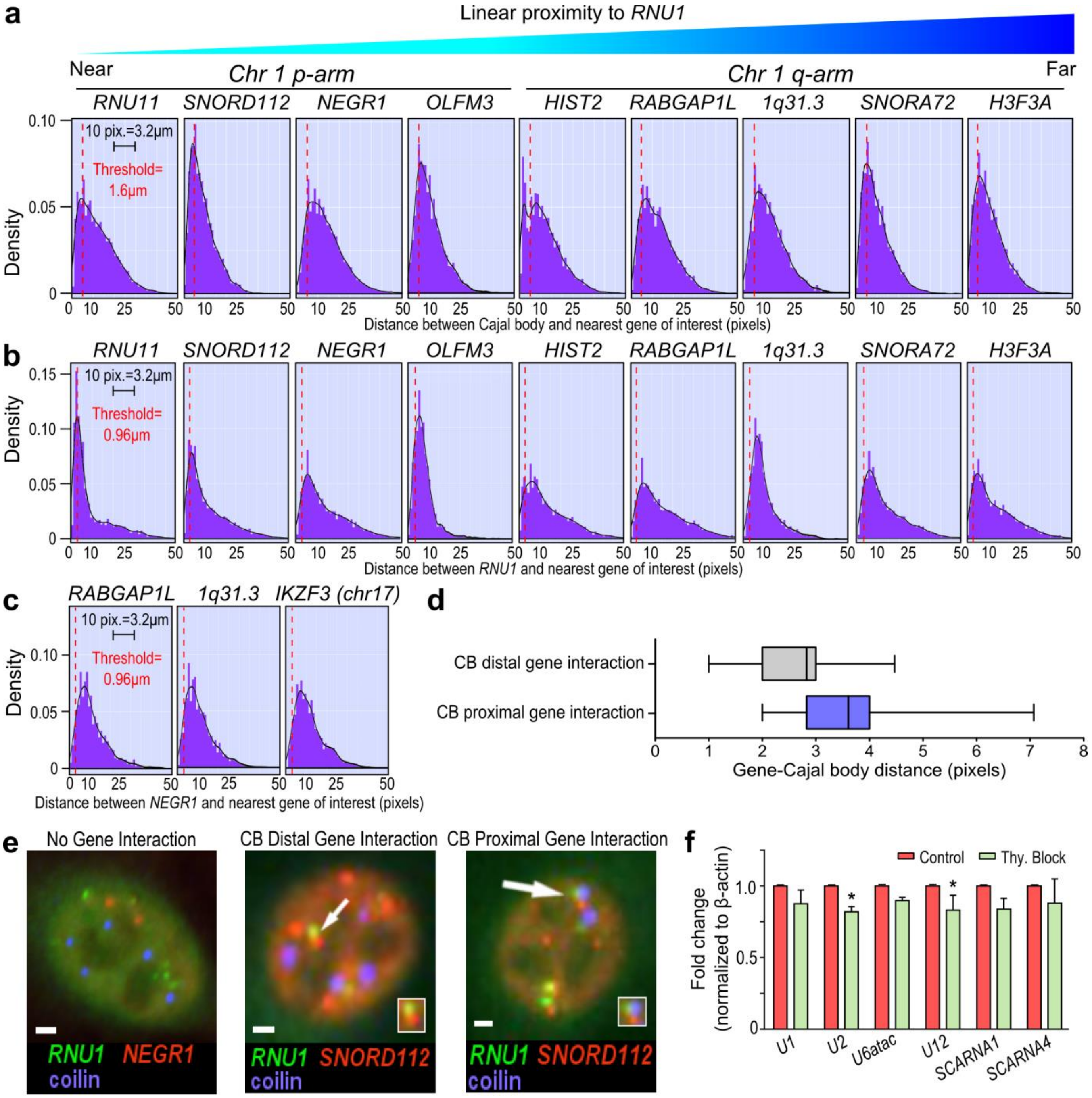

(a) Histograms of minimum CB-gene of interest distances (in pixels), (b) minimum *RNU1*-gene of interest distances, and (c) minimum *NEGR1*-gene of interest distances, following high-throughput image analysis detection of chromosome 1-localized DNA FISH foci and CB immunofluorescence. Blue bars = binned data, black line = estimated density function. Histogram generated by analysis of >1000 HeLa cells/DNA FISH probe, (d) Box-and-whisker plot of CB-independent (*RNU1*-gene of interest, grey) and CB-dependent (*RNU1* or gene of interest – CB detected by coilin immunostaining, blue) distances (pixels) quantified through manual scoring of >150 foci-foci interactions in HeLa cells. Error bars

represent SEM, (e) Representative microscopy images acquired using the Opera hi-throughput imaging system depicting HeLa cells displaying either a lack of gene interactions (left), a CB distal gene interaction between *RNU1* and *SNORD112* (center) or a CB proximal gene interaction between *RNU1* and *SNORD112* (right). White scale bar = 2μm, (f) Expression of U sn/snoRNA genes and scaRNAs in cells treated with 5mM thymidine to induce a cell cycle block by qRT-PCR. N=2, *=p<0.05, **=p<0.01.

Supplementary Figure 5

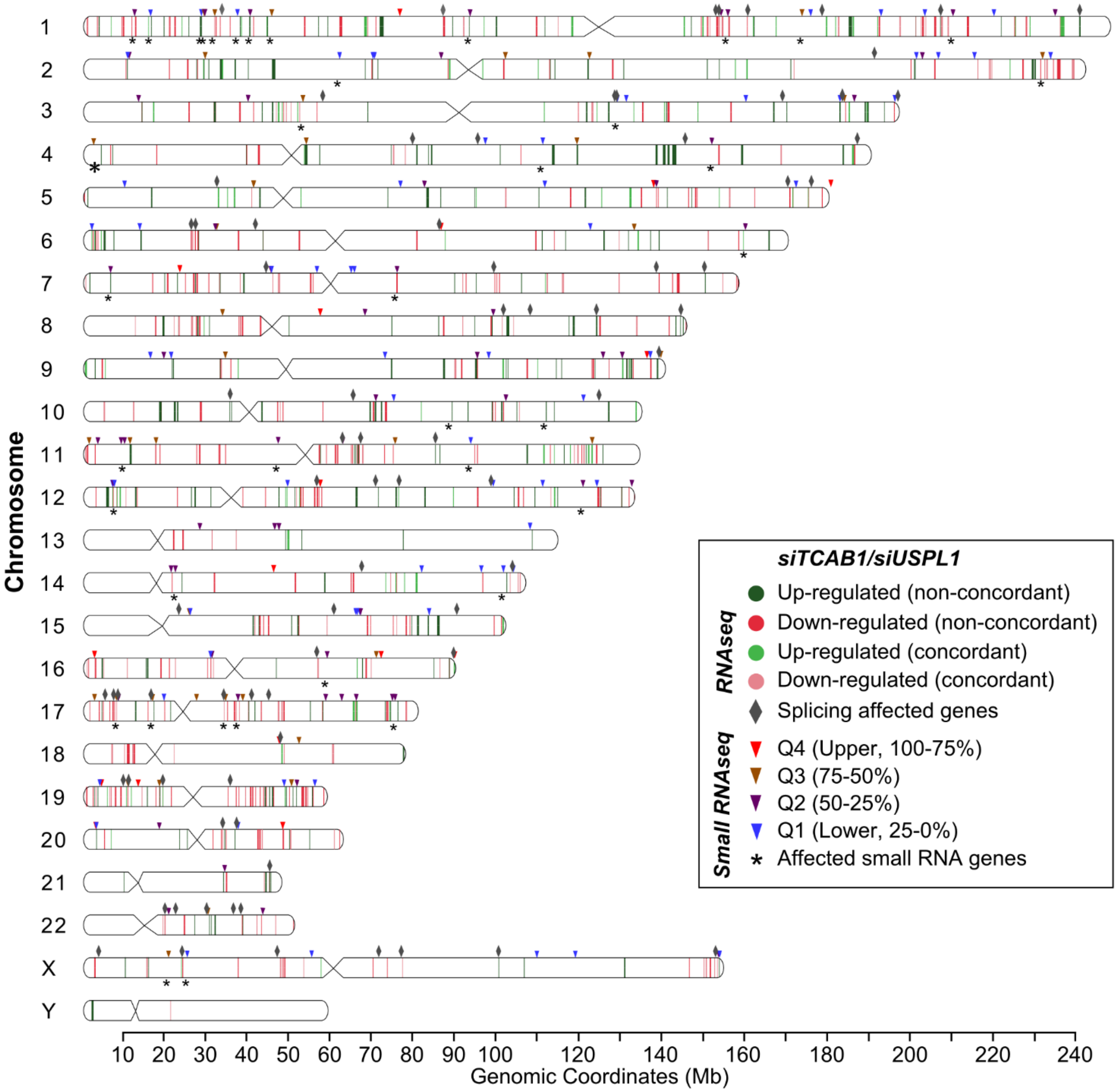

Karyogram depicting the locations of RNA expression changes, small U gene expression levels and alternative splicing events in HeLa cells following CB disassembly (siTCAB1/siUSPL1). Up-regulated genes (total RNA-seq) are denoted by green chromosome bands (non-concordant = dark, concordant = light green) and down-regulated by red (non-concordant = dark, concordant = light red). Baseline (siControl) expression of small RNA genes are denoted by triangles (red = Q4 (upper, 100-75%), brown = Q3 (75-50%), purple = Q2 (50-25%) and blue = Q1 (lower, 0-25%)) Grey diamonds = alternative splicing events, * = small RNA expression change. Figure was produced using Idiographica[1]

Supplementary Figure 6

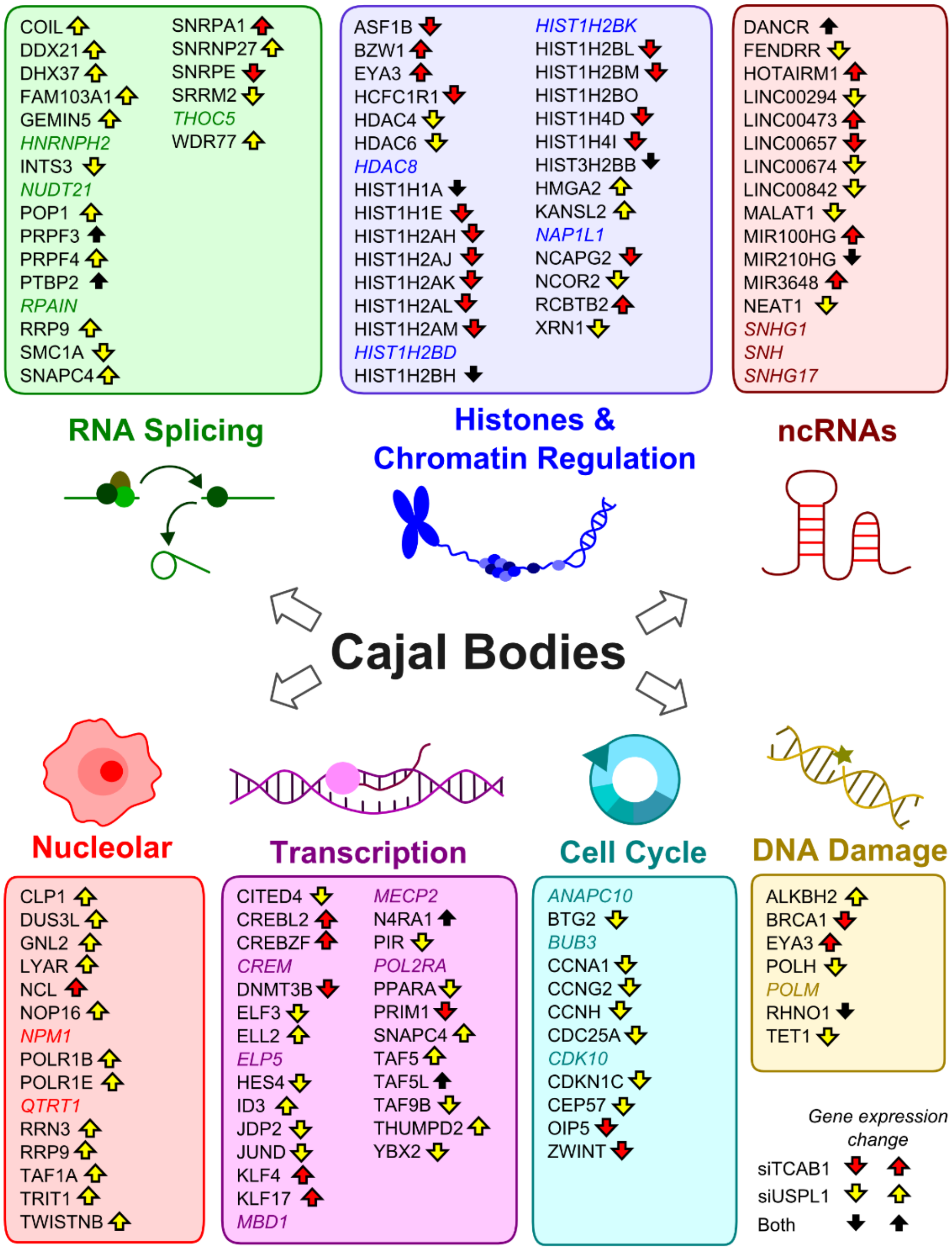

Represented here are seven nuclear-related functional groups of potential CB targets in HeLa cells which show a significant (p<0.01) reduction in RNA expression (identified by total RNA-seq) following TCAB1 or USPL1 siRNA treatment by CuffDiff or DEXseq analysis. Red or yellow arrows respectively identify TCAB1- or USPL1-only expression changes. Black arrows depict genes whose expression changed under both knockdown conditions. Genes that displayed alternative pre-mRNA splicing (assessed by Spanki and DEXseq) are italicized.

Supplementary Table 1

List of primers used in this study

Target	Forward	Reverse
H3F3A 4C bait	TAGCTCTTTATTGGAAAGAGTGATCT	TGCTTAAAACCAATACAACCACA
RNU1 4C bait	ATTGGAGTCCTTGTCAACTCTACA	ATGACAATCAGAGAAAGCTGA
MCAM	CTGCTGAGTGAACCACAGGA	TCAGGTCATGCAACTGAAGC
MUTYH	CTGTGATGGGATGATTGCTG	TCATCTTCTGCCCGTCTTCT
NSD1	GCCAATTGTTTGCACTTCCT	TCTGTGACTGGCTGTTCGG
PCNA	CTGAGGGCTTCGACACCTAC	TTTCCGTAACCGCTCATCTC
POLR2A Exon 22	AGGAAGGTGACTGCCAACAC	GTGAGCTTCCGGTCAGTCAT
POLR2A Exon 29	CTTCTTCCTCCCCCAAAAAC	TAGGTGCTCAGACCTCGTCA
POLR2A Exon 5	CGGAATGGAAGCACGTTAAT	GAGCCCTGCATCACAACAG
RBBP4	ATTCTTGCCACAGGATCAGC	CTGCATCTTCTGGGGATTGT
SF3B3	GAAATGGGTGGGACTGAGAA	GGCTTGACATGGCCAATACT
TPM2	GATGAAGCCAGYGCCAGAGYT	CGCACGTCAATGACATCACCT
BECN1	AGGAGAGACCCAGGAGGAAG	GTCAAAAAGGTCCCCAGTGA
BRCA1	GGAGTTGATCAAGGAACCTGTC	TGTCAAGCTGAAAAGCACAAAT
DHX8	ACCCTTGTACAACCGCTATGA	CCAGCAGGGAAGAAATGAGA
DMAP1	ACCTGAGCCTGGTCCTTCTT	GGGCCTCTTGAAAGTCAGTG
DUSP3	TAAAAACCCCACCATTTGGA	CTTCCCTGCTTGTCTTCTGG
EIF2B3	AGGCTGCTCTTCATGGCTAA	TGTTGTGAGGAAGCTGAGGA
EIF6	GGACATGGACTCGGTGGT	AACTGTGAGATCGGCTGCTT
EXOC7	TCACCTCAAGCCACCTTGG	AGCAGAGGACAGGCAAATG
EZH1	CTCAGTGCACACCCAACATC	TTCCAGCAAAAGGAAGCAGT
GNB1L	CTCCAAGCCTGCCTAGGTG	CCTTCCCCATCTCCCTTTAG
GPNMB	GCTTCAGCCATGTTGTGAAA	GCTGAAAGTATCCCGCCTCT
H3F3A	CATGGCTCGTACAAAGCAGA	GGGAAGTTTGCGAATCAGAA
HECTD3	GAGAACCTGGGTAGCGTGAA	CCGCTTTGGCATAAAGTTGT
HIST1H2AK	GGCTAAGACCCGTTCTTCAA	GATGATGCGGGTCTTCTTGT
HIST1H2BD	ATGGGGCTCATGAATTCCTTC	TTGGAACTGGTGTACTTGGTGA
HIST1H2BK	GCTCGTCTCAGGCTCGTAGT	TGATTCCCATGGCCTTAGAG
HIST1H3A	AAACAGACCTGCGTTTCCAG	GCTTGGACCGTCAGAGAGAC
HIST1H3E	GGTGCGAGAAATAGCTCAGG	CAAAACAATTCACGCCCTCT
HIST2H2AA3	CGGTCCTCGAGTATCTGACC	TCCGTCTTCTTAGGGAGCAG
HIST3H2A	AAATGTCCGGTCGTGGTAAG	CAGGATCTCGGCAGTCAAGT
HIST3H3	GAGCTGCTAATCCGCAAGTT	TGTCCTTAGGCATGATGGTG
Histone H3.2	GCTACCAGAAGTCCACGGAG	GATGTCCTTGGGCATAATGG
Histone H31.B	GCTGCTGATTCGCAAACTGC	GCATAGGTTGGTGTCCTCAAACAG
Histone H3C	CGCTGGTAAGCCTGTTTTT	CTCCGTGGACTTCTGTAGC
HMGB1	CTTCGGCCTTCTTCCTCTTC	CAGGCTTTCCTTTAGCTCGAT

HMGB1	CTTCGGCCTTCTTCCTCTTC	CAGGCTTTCCTTTAGCTCGAT
HNRNPH3	CGACCGGGACCATATGATAG	CTCCACCATAGCCATGTCCT
MAD2L2	TCACACGACAAGACCTCAACTT	CAGCGTGTCCTGGATACTGA
MCAM	CTGCTGAGTGAACCACAGGA	TCAGGTCATGCAACTGAAGC
MTOR	TTTGACAGAGTTGGAAGACAGT	AATTCTCCTATTGTTGCCAGGA
MUTYH	CTGTGATGGGATGATTGCTG	TCATCTTCTGCCCGTCTTCT
MYNN	TCACTGTCAGCTGTGAATAGCC	TTCCAACTGCAAGGAAACAAG
NBR1	GGCATTGAAATAACACCCAGA	CATTGCTCAATAGGTGCTGGT
NCAPH2	CTGTGTTCCTCCCCTCTG	TCTTGGCTCTAGGCTGGGTA
NOTCH1	GGCTACAACTGCGTGTGTGT	AGTTGGAGCCCTCGTTACAG
NPM1	TGCAGGAAGATGCAGAGTCA	TTTCTTCACTGGCGCTTTTT
PCNA	CTGAGGGCTTCGACACCTAC	TTTCCGTAACCGCTCATCTC
PSMC3IP	CCTGCCAGATATCAAACCTGA	AGTGTTGCCTGCATTTCTCC
RAB2A	GCAAGAATCCTTTCGTTCCA	GAGTCCATGTTCTCGTGCAA
RAN	TGTGTGGCAACAAAGTGGAT	AAAGCTGGGTCCATGACAAC
RBBP4	ATTCTTGCCACAGGATCAGC	CTGCATCTTCTGGGGATTGT
RNU1	GATACCATGATCACGAAGGTGGTT	CACAAATTATGCAGTCGAGTTTCC
RNU11	GTGCGGAATCGACATCAAGAG	CGCCGGGACCAACGAT
RNU12	AACTTATGAGTAAGGAAAATAACGATTCG	CGACCTTTACCCGCTCAAAA
RNU2	TTTGGCTAAGATCAAGTGTAGTATCTGTTC	AATCCATTTAATATATTGTCCTCGGATAGA
RNU3	GAACGTGTAGAGCACCGAAAA	TCAGACCGCGTTCTCTCC
RNU4	GGCAGTATCGTAGCCAATGAG	CCGTAGAGACTGTCAAAAATTGC
RNU5	GGTTTCTCTTCAGATCGCATAAATC	CTCAAAAAATTGGGTTAAGACTCAGA
RNU6	GCTTCGGCAGCACATATACTAAAAT	ACGAATTTGCGTGTCATCCTT
RNU7	GCATAAGCTTAGTGTTACAGCTCTTTTAGAA TTTGTC	CGTAGAATTCAGGGGCTTTCCGGTAAAAAG CCAG
RPS24	AAGGCGACAGTGCCTAAGAC	TCTCCAGCTCACTGTCTTGC
S100A4	CATCTGTCCTTTTCCCCAAG	TCTCTCCTCAGCGCTTCTTC
SCARNA1	GCACTTGATACTAACCGAGCTG	CTGGGCTTAAAAGACTCATGG
SCARNA2	GTGCAGGGTGAGTGTGAGTG	TCGCAGGAGGAGAGCTTTT
SCARNA4	CTGGAGGACTAAGAAGGCTGAG	GGTAGTACAGTCAGCCCTGTGATA
SCARNA7	TGTGTGCTTGTGGTGGCTA	TGGATCAGATCACAGGACCA
SF3B3	GAAATGGGTGGGACTGAGAA	GGCTTGACATGGCCAATACT
SNORA18	CTATCCCGATGGGGCTTT	TGTCTTGTAATTCCTTCCCACA
SNORA36B	CCAAAGTGTTAAGTTCAGTTCAGG	TGTAGGCTGGGGATAAATGAA
SNORA48	TCCCTGACCTGGGTAGAGTG	TGTCTTGTAATTCCTTCCCACA
SNORA55	AGCACCTGAATCTTTCCCATT	CTGTAGAGACAGGCCCCAAG
SNORA74B	AGAACCGTTCCTGTGATGGT	AGCAGCCAAAGTGAATGCTTA
SNORA78	ACTCTGGTTTCTCTTCAGATCGT	GTCGGAACAAGGCCTCAAA
SNORA7B	GGGATCGCATCTGGAGACT	CGCAGAGTGTCTTCCAATCA
STAT3	ACAGCCTCCTTGGTGCTTTA	TCTGCCCAGCCTTACTCACT
STAT5A	CTGAACAACTGCTGCGTGAT	GTGGACGATGACAACCACAG
TBP	TATAATCCCAAGCGGTTTGC	CACAGCTCCCCACCATATTC
TCAB1	AAGGCTGTAAGTCGGTCCT	GGGCTGAGGACATCAGAGAA
THOC5	TCAAACCTTTGCTGGCTCT	ATGGAGTGTTTGGGGAACTG

TP53BP1	CCAGAGCCTGAGGTTTTGTC	GCATCAGGAGAAGGAGCAAC
TPM2	GATGAAGCCAGTGCCAGAGT	CGCATCAATGACATCACCT
TUBA1A	ATTGGCAGCATCTTCTTTG	CCAGTGCCAAGTGACAAGA
TUBA3D	GCTTCCTCATCTTCCACAGC	CCATGAAGGCACAGTCAGAA
U4atac	GCGCATAGTGAGGGCAGTACT	GCACCAAAATAAAGCAAAAGCTCTA
U6atac	AGGTTAGCACTCCCCTTGACAA	TGGCAATGCCTTAACCGTATG
USPL1	TTCTGGAATGGCAGACCT	TTCGAGCACAGTCCAGTCAC
β-actin	TCCGGCATGTGCAAGGCCGGCTTCG	GTACTTCAGGGTGAGGATGCCTCTCTTG

Supplementary References

1 Kin, T. & Ono, Y. Idiographica: a general-purpose web application to build idiograms on-demand for human, mouse and rat. *Bioinformatics* **23**, 2945-2946, doi:10.1093/bioinformatics/btm455 (2007).
